# Tumor microenvironment as a novel therapeutic target for lymphoid leukemias

**DOI:** 10.1007/s00277-025-06237-w

**Published:** 2025-02-25

**Authors:** Shahrzad Mousavi, Soheil Nouri, Arezoo Sadeghipour, Amir Atashi

**Affiliations:** 1 Department of Hematology, Faculty of Medical Sciences, Tarbiat Modares University, P.O. Box 14115-111, Tehran, Iran; 2 Department of Biochemistry, Faculty of Biological Sciences, Tarbiat Modares University, P.O. Box 14115-175, Tehran, Iran; 3 Department of Medical Laboratory Sciences, Faculty of Allied Medical Sciences, Shahroud University of Medical Sciences, Shahroud, Iran

**Keywords:** Tumor microenvironment, Lymphoid leukemias, Anti-leukemic treatments

## Abstract

Lymphoid leukemias represent a significant global health burden, leading to substantial morbidity and mortality. The intricate interplay between leukemic cells and their surrounding tumor microenvironment (TME) is pivotal in disease initiation, progression, and therapeutic resistance. Comprising a dynamic milieu of stromal, immune, and leukemic cell populations, the TME orchestrates a complex network of signaling pathways and molecular interactions that foster leukemic cell survival and proliferation while evading immune surveillance. The crosstalk between these diverse cellular components within the TME not only fuels tumor progression but also confers resistance to conventional therapies, including the development of multi-drug resistance (MDR). Recognizing the pivotal role of the TME in shaping disease outcomes, novel therapeutic approaches targeting this dynamic ecosystem have emerged as promising strategies to complement existing anti-leukemic treatments. As a result, drugs that target the TME have been developed as complementary strategies to those that directly attack tumor cells. Thus, a detailed understanding of the TME components and their interactions with tumor cells is critical. Such knowledge can guide the design and implementation of novel targeted therapies for lymphoid leukemias.

## Introduction

Lymphoid leukemias cause significant morbidity and mortality worldwide. Morbidity and mortality vary in patients due to intrinsic factors like epigenetic changes and, also extrinsic factors such as signaling pathways, adhesion molecules, and cells that interact with the leukemic cells, that so-called tumor microenvironment (TME) [1].

A tumor microenvironment (TME) is a sophisticated mixture of both leukemic, and normal cells, including mesenchymal stem cells, fibroblasts, and immune cells, particularly T lymphocytes and macrophages [2]. Through several communication networks, including secreted substances, cell–cell junctions, and extracellular vesicles like exosomes, cells in the TME communicate with one another [3]. These interactions through different mechanisms contribute to tumor cells’ growth and progression.

The TME plays a significant role in neoplastic diseases. For the effective treatment of tumors, in addition to directly targeting tumor cells, it is also important to manage the microenvironment that contributes to resistance to anticancer therapies [4]. Tumor cells are initially responsive to therapy, but over time, many processes cause them to develop a drug-resistant (DR) phenotype, which makes them resistant to a variety of anticancer medications. MDR, or multi-drug resistance, is a significant obstacle in managing hematological malignancies [5].

In this review, we highlight the communications between leukemic, and normal cells, including stromal and immune cells in the bone marrow niches, signaling networks, and adhesion molecules that have a major effect on the regulation of TME. Then we discuss targeted therapies that may overcome the MDR situation in lymphoid leukemias. Finally, we will examine the targeting of components and signaling pathways involved in the tumor microenvironment, such as WNT signaling inhibitors, BCR inhibitors, immunomodulatory drugs, immune checkpoint blockade therapy, etc.

## Lymphoid leukemias

Lymphoid leukemias are divided into two categories: acute and chronic. These leukemias can be B-cell or T-cell neoplasms. Acute lymphoblastic leukemia (ALL) is the most common leukemia in childhood. It accounts for around 25% of all pediatric malignancies and is most common in children aged 2 to 5 years. Childhood ALL is caused by B-cell proliferation (B-ALL) in 85% of cases and T-cell progenitor cells (T-ALL) in only 15% [6]. B-ALL is characterized by a stop in lymphoid development and, as a result, the production of lymphoid cells as pro-B or pre-B [7]. B-ALL is distinguished by the expression of B-cell-specific antigens such as PAX5, CD19, CD20, CD22, CD24, and CD79a (found in the cytoplasm). CD20 is a typical mature B-cell antigen, but leukemic lymphoblasts may be weak or not positive at all [7]. T-cell ALL accounts for 15% of childhood ALL cases and 25% of adulthood ALL cases. A common risk factor for T-ALL, infiltration of the central nervous system (CNS), continues to be a barrier to long-term remission [6].

CLL is one of the most frequent leukemias in the Western world, making up 37% of cases, with a male-to-female ratio of 1.9 and an average age at diagnosis of 70 and older [8]. The disease is characterized by the clonal development of leukemic B cells and can be identified by the presence of CD5, CD19, and CD23, as well as apoptosis and quiescence deficits in a large number of CLL cells [9]. A small percentage of cases progress quickly to advanced stages, frequently with negative outcomes. The majority of individuals have an indolent condition that may not even need therapy [10].

## Tumor‑associated stromal and immune cells

**Fig. 1:**
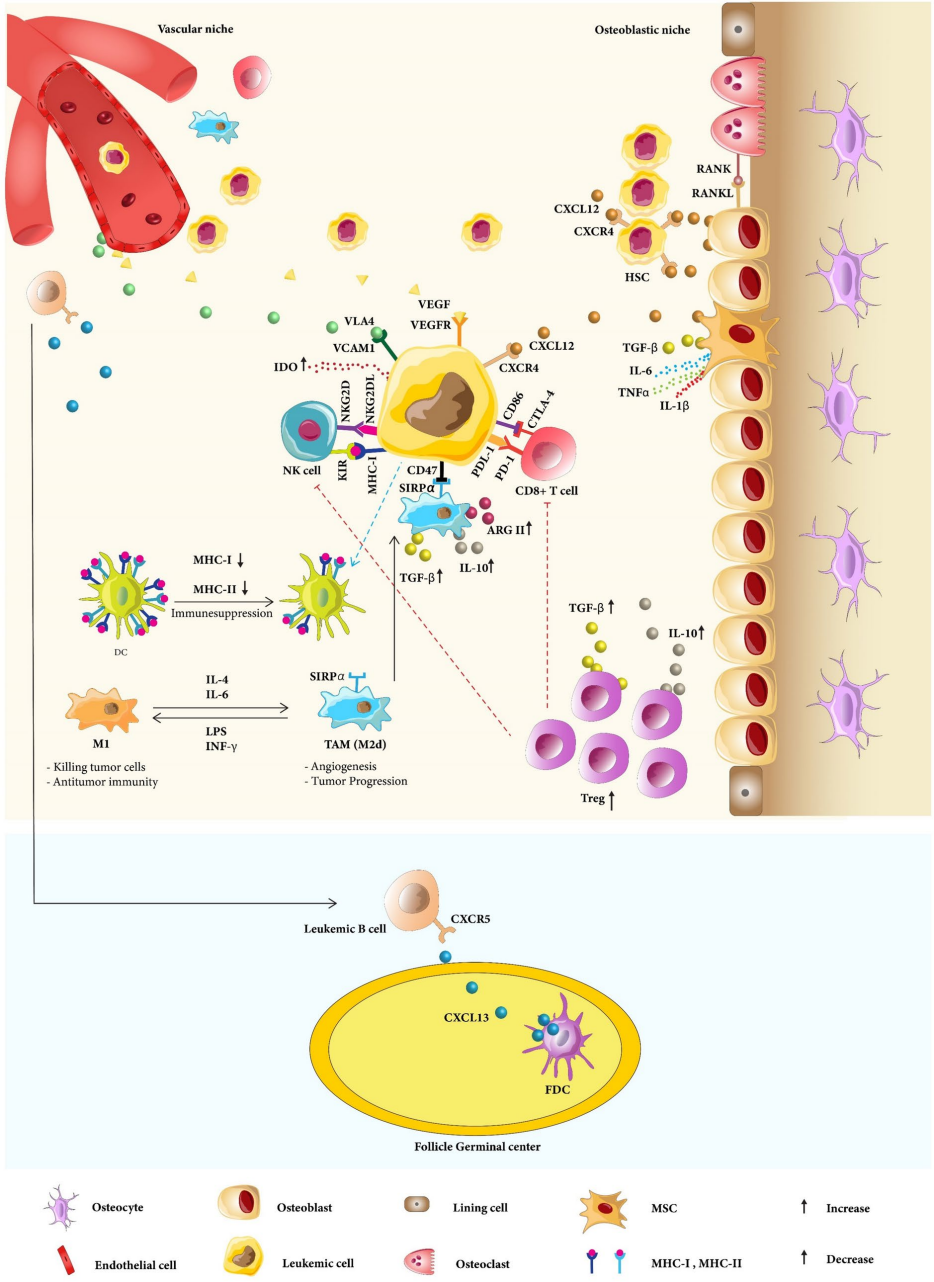
Cancer cells secrete IDO that reduces T cell cytotoxicity and enhances Treg-mediated immunosuppression. Tregs by secreting IL-10 and TGF-β negatively control NK cells and cytotoxic T cells’ responses. Moreover, interactions between inhibitory molecules like CTLA4 and PD1 on T cells with their ligands on tumor cells help immunosuppression of the TME. Tumor-associated macrophages (TAMs) are an M2-like phenotype and release TGF-β, IL-10, arginase, and metalloprotease to help suppress the immune system. Additionally, cancer cells express more CD47 that interacts with SIRPα on a phagocyte and cause phagocytosis suppression. MSCs have abnormal CXCL12 or TGF-β production and boost the production of pro-inflammatory mediators like IL-1, IL-6, and TNFα. CXCL12 binds to CXCR4 on leukemic cells. Endothelial cells exist in the vascular niche and produce VEGF and VLA4 that bind to VEGFR and VCAM1 on leukemic cells, respectively. FDCs secrete CXCL13, which drives B cells to the germinal center’s light zone

Leukemic cells interact with different types of cells, including stromal cells such as Mesenchymal stem cells (MSCs), fibroblasts, and endothelial cells, and immune cells such as T-cells, tumor-associated macrophages (TAMs), and dendritic cells (DCs). To date, the majority of cancer therapies target tumor cells directly. Since, immune and stromal cells in the TME are genetically stable, unlike tumor cells, they are more likely to be resistant to MDR mechanisms, so targeting the TME is now an effective therapeutic approach in addition to killing tumor cells directly [11]. According to experimental data, the immune system has a dual role in cancer: In the process of cancer immunoediting, tumor suppression may occur in the elimination phase by utilizing innate and adaptive immunity working together to destroy developing tumors. On the other hand, tumor development may occur in the escape phase if immune cells do not respond to the tumor cells. Tumor immune escape describes the process by which tumor cells proliferate and spread by evading detection and assault from the immune system through different mechanisms, representing a crucial tactic for tumor persistence and progression. The escape phase may occur due to various mechanisms, including antigen loss in which the tumor cells are no longer recognized by adaptive immunity, insensitivity to immune effector mechanisms, or induction of an immunosuppressive state within the TME by tumor cells (Fig. 1) [12].

### Mesenchymal stem cells (MSCs)

Mesenchymal stem cells were the name used for these cells by Caplan et al. in 1991 [13]. Due to their unique characteristics, MSCs are usually purified by plastic adherence and remain highly heterogeneous after isolation, yielding a diverse cell population with varied stemness potential. To avoid confusion, the word "stem" in "mesenchymal stem cell" has been substituted by "stromal," which refers to a bulk population with secretory, immunomodulatory, differentiation, and homing features [14]. BM-MSC are located in osteoblastic and perivascular niches. When a tumor exists, various signals (growth factors, cytokines, etc.) allow communication between the tumor and BM niches. Lagneaux et al. showed that MSCs taken from CLL patients have unique characteristics that set them apart from healthy MSCs. For instance, these cells produce aberrant amounts of CXCL12 or TGF-β, two substances that are necessary for the survival of leukemic cells, and they also exhibit abnormal cell growth as a result of an increase in apoptotic cell death [15].

Beneforti et al. discovered that the pro-inflammatory cytokines, such as IL-1β, tumor necrosis factor-α (TNF-α), and IL-6 which are released in response to different sorts of infections [16], work with BM-MSCs to help ETV6-RUNX1-expressing cells find a suitable niche and become more susceptible to transformation through elevated DNA damage [17]. The expression of *ETV6-RUNX1* in human cord blood progenitor cells reportedly caused the expansion of a candidate preleukemic population that had a growth advantage in the presence of transforming growth factor-beta [18]. MSCs that have been isolated from B-ALL patients’ BMs (ALL-MSCs) displayed diminished hematopoiesis maintenance and proliferative potential [19]. When compared to healthy donor-MSC, ALL-MSC proliferative ability was diminished because it had a negative correlation with the amount of BMP4 (Bone Morphogenetic Protein 4) [20]. The capability of ALL cells to generate immunosuppressive dendritic cells (DCs) and to polarize macrophages to an M2-like pro-tumorigenic phenotype is enhanced by BMP4 overexpression, which is relevant to the actions that may promote leukemia [21]. MSCs can aid in the release of inflammatory substances in the BM niche alongside B-ALL cells. MSCs isolated from the BM of B-ALL patients were found to have enhanced nuclear translocation of the NF-kB transcription factor, which boosted the synthesis of pro-inflammatory cytokines such as IL-6, IL-1, and TNF-α, as well as interferon type I and type II [22].

### Follicular dendritic cell (FDC)

FDCs are immune cells typically found in secondary lymphoid organs, including the spleen and lymph nodes [23]. However, FDCs have been seen in BM infiltrates in patients with CLL [24]. Secondary lymphoid organs play a crucial role in the immune system by serving as sites for the activation and proliferation of lymphocytes. These organs filter bodily fluids and help mount immune responses against pathogens. The primary secondary lymphoid organs include the spleen, Lymph Nodes, etc. Primary follicles are specialized structures found within secondary lymphoid organs. Germinal centers represent a critical phase in the adaptive immune response that evolves from primary follicles following antigenic stimulation, composed of dark zone and bright zone, in which FDCs are found in the bright region of germinal centers and the center of primary follicles [25]. FDCs can capture antigen–antibody complexes on the cell surface through the stimulation of complement receptors 1 (CR1 or CD35) and 2 (CR2 or CD21) [11] and present untreated antigens to B cells [26]. Due to their cytokine release, their ability to activate BCR signaling, the adhesion molecules they carry, and their protective effects on CLL-B cell survival, FDCs are another important part of the stromal microenvironment (Fig. 1) [27].

### Tumor-associated macrophage/Nurse-like cell

Macrophages divide into two phenotypes, M1 and M2. LPS and IFN-γ polarize M1 macrophages. They produce proinflammatory cytokines, have high levels of iNOS, and express inflammatory chemokines including CXCL9 and CXCL10. IL-4 and IL-6 polarize M2 macrophages. They have high levels of Arg1 expression and release TGF-β, IL-10, arginase, and metalloprotease to help suppress the immune system [28]. In response to TME signals, a dynamic process known as "macrophage polarization" that converts M1 into M2 takes place [29]. Although neither M1 nor M2 macrophages exhibit a specific pattern of gene expression, these cells usually exhibit large amounts of the expression of several genes. For instance, the macrophages M1 and M2 are typically identified by the markers iNOS and Arg1, respectively. M1 macrophages typically have anticancer effects, while M2 macrophages typically have pro-tumor effects [30].

Tumor-associated macrophages (TAMs) can produce chemokines, cytokines, and growth factors to recruit immunosuppressive cells and promote tumor growth [31]. TAMs are thought to be an M2-like phenotype [32]. The role of M2 macrophages in cancer is multifaceted, primarily characterized by their ability to create an immunosuppressive environment that fosters tumor growth and metastasis. Their involvement in angiogenesis, immune evasion, and drug resistance makes them a critical target for therapeutic interventions aimed at improving cancer treatment outcomes. Studies have shown that macrophage infiltration into BM and spleen increases during the early stages of leukemia progression and decreases thereafter in non-irradiated mouse T-ALL models [33]. A potential therapeutic approach is the repolarization of M2-like macrophages to the M1 phenotype (Fig. 1) [34].

In chronic lymphocytic leukemia (CLL), macrophages are referred to as "nurse-like cells" (NLCs), which share a phenotype with TAMs in B-cell lymphoma [35]. Ex vivo culture of CLL cells results in rapid apoptosis, whereas co-culture with normal cells present in the CLL microenvironment, such as bone marrow-derived MSCs (BM-MSC) or monocyte-derived nurse-like cells (NLCs), leads to CLL cell survival [36].

Furthermore, ALL and other hematologic and solid malignancies commonly overexpress CD47 [37]. CD47 is a self-reactive protein found in all normal cells that regulates cell processes such as cell migration, cytokine generation, and T-cell activation [38]. The ability of CD47 to regulate innate immune surveillance when linked to the membrane protein SIRPα on macrophages and other myeloid cells has recently gained a lot of attention [39]. When CD47 on the target cell interacts with SIRPα on a phagocyte, phagocytosis is suppressed [40].

### T-cell and dendritic cell

Cancer cells produce immunosuppressive ligands to suppress immunological checkpoints, such as programmed death ligand 1 (PD-L1) and PD-L2, as well as other ligands, which prevent effective T-cell killing functions [41]. CD28-CD80/CD86 activation signaling is suppressed by cytotoxic T lymphocyte-associated protein 4 (CTLA-4). CTLA-4 is an inhibitory checkpoint on T cells that binds to CD80/86 ligands with higher affinity than CD28. This binding inhibits activation signaling and prevents uncontrolled proliferation of activated T cells [42]. CTLA-4 regulatory actions predominantly limit CD4 + T cell activation while promoting regulatory T cells (Tregs), resulting in a pro-tumor immunosuppressive phenotype [43]. Furthermore, PD-1 (CD279) interacts with PD-L2 and PD-L1 to block immune responses [44]. The engagement of PD-1 with either ligand leads to inhibition of T-cell activation, which is a mechanism exploited by tumors to evade immune detection. The interaction of PD-1 on tumor-infiltrating lymphocytes (TILs) with PD-L1 and PD-L2 is a significant mechanism of tumor immune evasion, and so, a novel therapeutic target. Additionally, the strong presence of PD-1 and its ligands on tumor cells and TILs indicated that blocking this route could result in less severe immunological toxicity toward healthy cells than CTLA-4 blockage [45]. Adverse events associated with CTLA4 blockade are generally colitis, diarrhea, pruritis, dermatitis, and fatigue.

Co-culture of CLL cells in combination with allogeneic T cells or autologous tumor-associated T cells results in a negative feedback loop in which inhibitory receptors and ligands are considerably increased in reaction to anti-tumor T cells, inhibiting T-cell immune synapse signaling and effector functions [46]. The aberrant CTLA-4 upregulation seen in T cells from CLL patients was connected to a higher proportion of Tregs and an advanced Rai stage [47]. The Rai staging system classifies CLL into three separate risk groups and five stages. In stages 3 and 4, which are the advanced stages, an abnormal increase in the number of lymphocytes in the circulating blood and the marrow, anemia (hemoglobin < 11 g/dL), an abnormal increase in the number of lymphocytes in the circulating blood and the marrow, and thrombocytopenia (platelet counts < 100,000/uL) occurs. In their normal function, Tregs control immune responses to maintain homeostasis and autoimmune tolerance. However, their aberrant function can suppress the immune response of the TME, which ultimately causes tumor growth [48]. Studies have shown that primary T cells co-cultured with CLL-derived CTLA-4 + Mec1 cells produced less IL-2, demonstrating that CTLA-4-expressing leukemic cells hindered T cell co-stimulation [42]. According to another study, in CLL patients PD-1 presence was raised on both CD8 + and CD4 + T cells, and interaction between PD-1 on CD8 + T cells and PD-L1 on CLL resulted in lower IFN-γ production (Fig. 1) [49].

Moreover, IDO (indoleamine 2,3-dioxygenase) is a cancer cell-secreted immunosuppressive modulator that reduces T cell cytotoxicity, TNF-α, IFN-γ, and IL-2 production and enhances Treg-mediated immunosuppression, resulting in the development of a tolerogenic environment at tumor locations [50].

Dysfunction in tumor-infiltrating DCs is caused by immune or leukemic cells that express PD-L1, PD-1, CTLA4, and TIM3. NF-kB activation is essential for DC functions such as costimulatory molecule expression, presenting of antigens, and release of cytokines which leads to T cell inactivation and is inhibited specifically by PD-1 expression [51].

## Signaling networks

### BCR signaling

The multivalent interaction with the B cell receptor (BCR) complex initiates a cascade of biochemical reactions that lead to the activation of transcription factors and the regulation of gene expression [52]. B cell activation is triggered by the binding of a ligand (known as an antigen) to the BCR. To obtain BCR activation, clustering of the BCR element at the plasma membrane as well as phosphorylation of the ITAM in the cytoplasmic tail of CD79A and CD79B by Lyn and other Src family kinases (Fyn, Blk) are required. These events activate downstream signaling pathways including SYK, BTK, PI3K, MAP kinase and RAS activation, protein kinase Cβ (PKCβ), phospholipase Cγ, and NF-kB signaling [53, 54]. Many of these processes are upregulated in normal B cells and aberrantly activated in B cell malignancies, including CLL. Disruption of the BCR signaling pathway in CLL is characterized by active phosphorylation of specific kinases, including BTK, SYK, and PI3K, and variable response to IgM stimulation [55].

BTK (Bruton Tyrosine Kinase) is expressed in hematopoietic cells, especially B cells [56]. After BCR activation, BTK is activated by other tyrosine kinases such as Lyn and SYK, activating transcription factors required for B-cell proliferation and differentiation [57]. SYK (Spleen Tyrosine Kinase) activates signaling pathways downstream of the BCR. Syk-deficient mice have severely impaired B-lymphopoiesis [58], blocking the transition from Pro-B to Pre-B, consistent with a critical role for Syk in signaling from pre-B cell receptors. In addition, current in vivo studies have shown that SYK is necessary for the survival and maintenance of mature normal and malignant B cells [58]. PI3K (Phosphoinositide-3-kinase) integrates and transduces signals from surface molecules such as the BCR [59], chemokine receptors, and adhesion molecules, thereby regulating cellular functions such as cell growth, survival, and migration [60].

### WNT

WNT proteins can activate a variety of different pathways that are often classified as β-catenin-dependent (Wnt/β-catenin) and independent pathways (Wnt/PCP) [61]. Since cell proliferation, cell cycle regulation, and stem cell homeostasis are all closely related to the Wnt/β-catenin pathway, its dysregulation is prevalent in many malignancies [62].

The protective actions of MSCs against B-ALL cells require the activation of the WNT pathway. WNT signaling elements are produced at a higher level in B-ALL cells than in normal cells [63]. Notably, ALL is characterized by abnormal activation of WNT-β-catenin-dependent genes, such as TCF1, C-MYC, AXIN1, and LEF1 [64]. An in vivo mouse model of T-ALL demonstrated inactivation of PTEN and overexpression of c-Myc, resulting in increased expression of β-catenin in leukemic stem cells [61]. Furthermore, abnormal stimulation of WNT/β-catenin signaling is currently suggested as a chemoresistance mechanism in ALL [65].

**Fig. 2:**
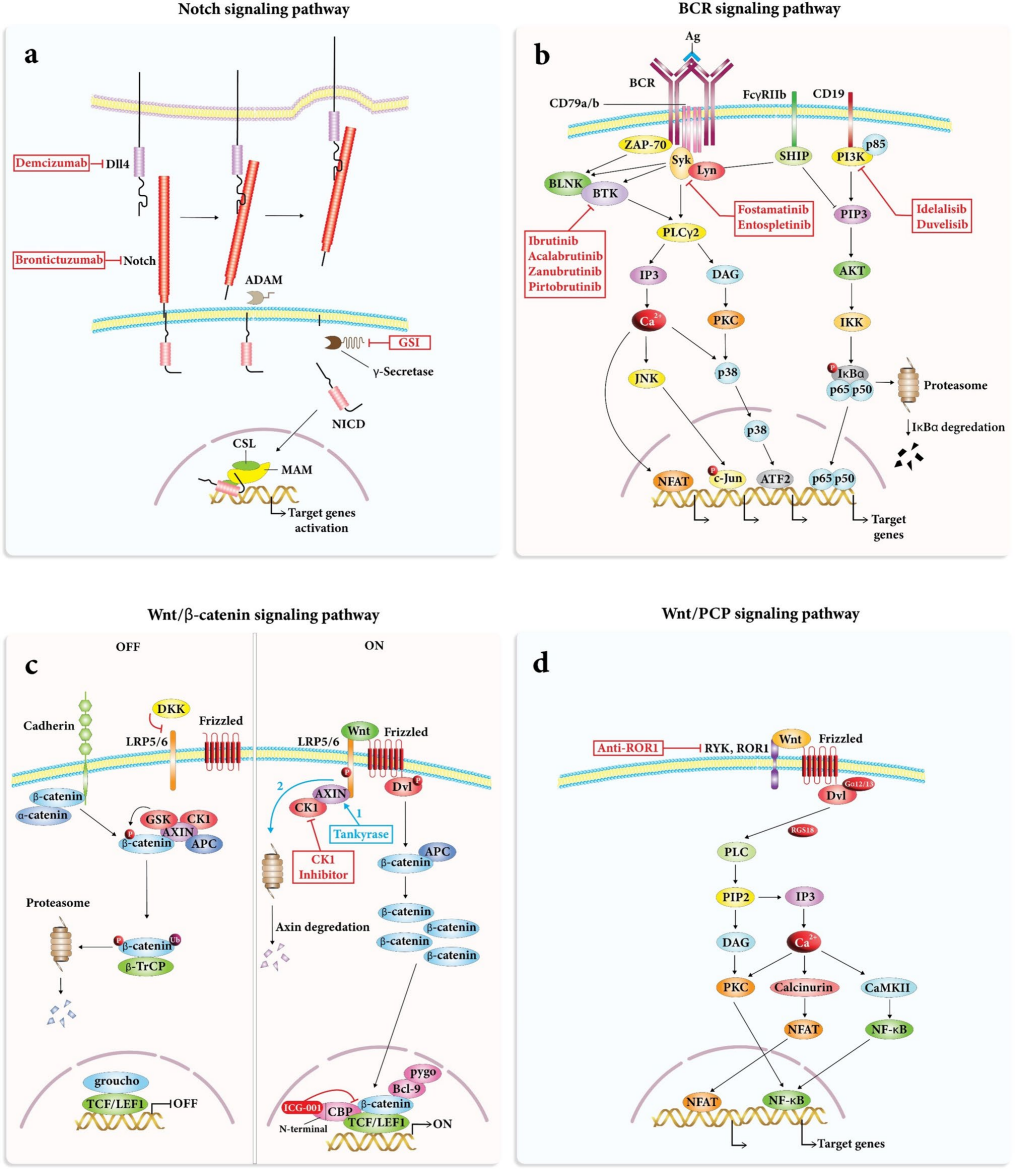
**a** NOTCH binds to its ligands, such as JAG1-2 or DLL1-4. Then proteolytic cleavages of the extracellular domain by the TACE, followed by γ-secretase happen. This causes the release and nuclear translocation of the ICN1. **b** Binding of antigen to the BCR induces phosphorylation of ITAM in the cytoplasmic tails of CD79A and CD79B by Lyn and other Src family kinases (Fyn, Blk). This activates downstream signaling pathways including SYK, BTK, and PI3K. **c** Wnt ligands are secreted proteins that bind to Frizzled receptors on the cell surface, initiating the signaling cascade. Second, upon activation, Disheveled proteins inhibit the activity of a protein complex called the destruction complex, which generally targets β-catenin for degradation. This leads to the accumulation and translocation of β-catenin into the nucleus. In the nucleus, β-catenin binds to T-cell factor/lymphoid enhancer factor (TCF/LEF) transcription factors, forming a transcriptional complex that regulates the expression of target genes. **d**) Wnt ligands bind to Frizzled receptors, which then activate ROR1. Activation of ROR1 leads to the activation of downstream signaling pathways

ROR1, a Wnt-5-dedicated receptor, is present on the surface of CLL cells but not on mature B-cells [66]. ROR1 is a sensitive marker that may be used to evaluate minimal residual disease (MRD) in CLL remission patients [67]. Wnt5a-ROR1 interaction has been shown to support TCF3-PBX1 cell proliferation by activating the STAT3 and AKT-PI3K pathways [68]. The WNT/β-catenin pathway activates LEF1 which regulates the expression of its target genes [69, 70]. LEF1 is essential for B-cell growth in its early stages [71]. Experimental LEF1 silencing decreased CLL cell survival but did not affect the viability of healthy CD19 + B cells [72]. CK1 is a substance that phosphorylates numerous WNT network targets. In primary CLL relative to healthy peripheral blood B-cells, CK1 expression was found to be higher [73]. There is evidence that CLL cells are susceptible to CK1 inhibition, which disrupts CLL cell polarity, blocks chemotaxis in vitro in primary CLL cells and CLL-derived cell lines, and reduces primary CLL cell homing in vivo in a xenograft mouse model (Fig. 2) [73, 74].

### Notch

The surface receptor protein NOTCH binds to its specific ligands, such as Delta-Like 1–4 (DLL1-4) and Jagged 1 and 2 (JAG1-2). This boosts proteolytic cleavages of the extracellular domain by the TACE, followed by γ-secretase-mediated cleavage that promotes the release and nuclear translocation of the NOTCH intracellular domain (ICN1). Then ICN1 in the nucleus attracts coactivators to create a transcription-activating complex that eventually mediates its actions (Fig. 2) [75].

Since around 60% of T-ALL patients have a NOTCH1 mutation, ALL is strongly linked to Notch signaling dysregulation [76]. Furthermore, Notch signaling may have a role in chemotherapy resistance [77], implying that Notch suppression could be used as a targeted therapeutic method. Notably, Notch signaling promotes crosstalk between leukemic cells and the bone marrow niche in B- and T-ALL patients. Actually, in Notch-dependent T-ALL models, tumor cells suppress proper hematopoiesis, leading to aberrant B cell differentiation and myeloid cell growth [78]. Contrary to the recognized involvement of the Notch network in T-ALL, almost nothing is known about its function in B-ALL. Notch promotes apoptosis and survival resistance in B-CLL cells and triggers apoptosis and proliferation arrest in mature B-cells [79].

## Adhesion molecules

Tumor cells migrate to tissues due to their attraction to chemokines such as CCL19 and CCL21, released by high endothelial venules, as well as CXCL12 secreted mostly by stromal and NLCs; and CXCL13 of FDCs. These chemokines interact with receptors on the tumor cells. Adhesion molecules like VLA-4 and their corresponding binding partners such as VCAM1 aid in the migration and targeting of tumor cells by immune attacking cells [80].

### CXCL12-CXCR4

CXCL12 (or stromal cell-derived factor-1, SDF-1) is secreted by BMSCs (bone marrow stromal cells) that binds to its receptor on leukemic cells, the C-X-C motif chemokine receptor 4 (CXCR4, or CD184). CXCR4 activation causes upregulation of various signaling networks including PI3K/AKT, PKC, MEK/ERK, and p38 MAPK [81]. Furthermore, MEK/ERK and p38 MAPK kinase promote T-ALL migration via interaction with the α2β1 integrin, indicating that blocking this pathway may be a promising target for T-ALL treatment [82]. In addition, CLL B cells express more CXCR4 than normal B cells, resulting in enhanced responsiveness to CXCL12 [83]. An in vivo investigation confirms this, revealing that higher CXCR4 levels are linked to an elevated probability of lymphoid organ invasion (Fig. 1) [84].

### VLA4-VCAM1

VLA-4 (CD49d) is an α4β1 integrin that is present on cytokine-activated endothelium and binds to Fibronectin (FN) and vascular cell adhesion molecule-1 (VCAM-1/CD106). VLA-4 participates in lymphocyte trafficking and recruitment and is present in the majority of hematopoietic cells including monocytes, and lymphocytes [85]. Patients who exhibited high VLA-4 activity at their first relapse had a considerably worse reaction to chemotherapy compared to the first chemotherapy, as well as a lower event-free survival and overall survival [86].

In CLL cell attachment to stromal cells, VLA-4 integrins work in conjunction with chemokine receptors [87, 88]. The interaction of leukemia and stroma via VCAM-1/VLA-4 binding results in the bidirectional stimulation of nuclear factor kappa B (NF-kB) mediated molecular networks, which is necessary for the development of chemoresistance both in vitro and in vivo [89].

The VLA-4/VCAM-1 axis promotes pro-survival signaling networks in ALL cells, which therefore causes chemoresistance. In MSC-leukemia co-cultures, VCAM-1 inhibitor could reestablish chemosensitivity to cytarabine and etoposide [90], indicating that targeting VLA-4/VCAM-1 may be a useful method to reduce stroma-mediated chemoresistance in B-ALL [91].

### CXCR5-CXCL13

The CXCR5/CXCL13 axis is critical in CLL recruitment and trafficking because CXCR5 is elevated on the CLL B-cell surface while CXCL13 is generated by stromal cells in B cell regions of secondary lymphoid organs. [92]. FDCs secrete CXCL13, which drives B cells to the germinal center’s light zone [93]. Using the E-Tcl1 CLL mouse model, Heinig et al. demonstrated that CXCR5 deletion lowers E-Tcl1 leukemogenesis and CLL proliferation. As a result, this chemokine was found to be required for CLL cell attraction into the germinal center [23].

## Hypoxia

Rapid tumor growth reduces normal oxygen levels from 2–9% to under 2% in bone marrow niches [94]. Several research indicate that hypoxia facilitates a sequence of changes in the TME that promote clonal spread from diverse cancer cells and fatal phenotypes. A hypoxic response is mainly induced by hypoxia-inducible factor-1α (HIF1-α) [95]. Elevated levels of HIF-α were identified in 67% of childhood ALL, and 100% of B-CLL [96]. In a hypoxic environment, HIF1-α activation reduces E-cadherin expression and boosts the function of Snail and Twist, two transcription factors that promote epithelial-mesenchymal junction (EMT). EMT is a procedure associated with the process of chemotherapy resistance [97]. Hypoxia is also related to lower extracellular pH in TME, inhibits humoral and cell-mediated immune responses, and induces DR by upregulating the transporter protein P-glycoprotein [98].

## Exosomes

Exosomes are nano-sized extracellular vesicles (EVs) that play a key role in tumor cells and TME interactions. Tumor proliferation affects oxygen levels, causing hypoxia and a greater secretion of EVs like exosomes by tumor cells [99]. Recent research has indicated that TME-released exosomes play a significant role in metastasis, tumor growth, and MDR because of their capacity to interact between tumor cells and TME [3]. Tumor-associated cells have been demonstrated to release more exosomes than normal cells [100]. It has been estimated that this amount is twice that contained in healthy human blood [101]. Exosomes are multi-signaling mediators that aid in intercellular communication, drug trafficking, and the transport of particular chemicals to target cells [102]. Several resistance mechanisms are primarily regulated by miRNAs found in the tumor cell-driven exosomes. These processes include drug target downregulation, DNA repair, apoptosis, cell cycle regulation, decreased concentration of anticancer drugs, and modulation of TME [103].

MiRNAs are essential molecules present in exosomes. MiR-508-5p and miR-324-3p, both regulate ABCA3 gene expression and participate in ALL resistance mechanisms. In childhood ALL patients, this gene generates an ABC transporter that induces drug efflux from cancer cells [104]. MiR-1246, miR-1308, miR-1228, miR-149, and, miR-455-3p are more abundant in the exosomes of ALL cell lines [105]. Exosomes produced from CLL have been shown to transport miRNAs to stromal cells with cancer-associated fibroblast features, increasing CLL progression. Notably, miR-146a was shown to be 15 times more abundant in CLL exosomes than in healthy cells, and it has been demonstrated to activate the AKT signaling network and NF-kB [106].

## Epigenetic mechanisms

Epigenetics now refers to chromatin-based reactions that control DNA-templated processes. Epigenetic control primarily involves changes in DNA modifications, chromatin remodeling, histone modifications, and noncoding RNAs. Epigenetic control is critical in many DNA-based functions, including repair, transcription, and replication [107].

DNA methylation is defined as the addition of a methyl group to the carbon-5 position of cytosine to generate 5-methylcytosine (5mC). DNA methylation is often performed at cytosine-guanine dinucleotide (CpG) sites by DNA methyltransferase (DNMT) enzymes [107]. Two components of the altered DNA methylation in hematologic malignancies are often observed: the methylation level of the methyltransferase genes themselves, as well as mutations in either the methyltransferase or demethylase genes. Tirdad et al. discovered that while the control group displayed a relatively methylated promoter in the DNMT1 gene, patients with B and T-ALL had unmethylated promoters [108]. In B-chronic lymphocytic leukemia (CLL), alterations in the methylation patterns of the methyl-binding proteins MBD2 and MeCP2 were noted [109]. Prognosis and treatment effectiveness for patients with ALL and AML may be predicted by the recurrent alteration in their DNA methylation profile [110].

## The therapeutic target of lymphoid leukemias

### Proteasome inhibitors

The proteasome, a key component of the ubiquitin–proteasome system (UPS), plays a multifaceted role in cancer biology. It is primarily responsible for degrading misfolded or excess proteins, thus maintaining cellular protein homeostasis. However, in cancer cells, the proteasome’s functions can become dysregulated, contributing to tumorigenesis and influencing treatment responses. Studies have revealed that Proteasome inhibitors can cause programmed cell death in solid tumors, Burkitt’s lymphoma cells, and leukemia cell lines [111]. Bortezomib has been shown to cause apoptosis and prevent the effects of cytokines on models of ALL [112]. IKKβ and RIP2 are two NF-kB upward activating kinases that are activated by the drug to produce a non-proteasomal breakdown of IkB and enhance NF-kB DNA binding [113]. Additionally, by decreasing the angiogenic pathway while promoting osteoblastogenesis activity, it has an impact on medullary function [114]. According to the most recent research, the drug might be useful in ALL relapsed and resistant pediatric patients when used with reinduction chemotherapy based on combinations with pegaspargase, dexamethasone, vincristine, or mitoxantrone (Table 1) [115].

**Table 1:** List of drugs or compounds targeting TME

Type	Compound/drug	Target	Function	Clinical trial	Reference
Proteasome inhibitors	Bortezomib	Proteasome	Produce a non-proteasomal breakdown of IkB and enhance NF-kB DNA binding	−	[112]
	Carfilzomib	Proteasome	A proteasome inhibitor	−	[95]
Immune Checkpoint Blockade Therapy (ICB)	Nivolumab	PD1	Anti-PD1	NCT01822509	
	Pembrolizumab	PD1	Anti-PD1	NCT02332980	
	Atezolizumab	PDL1	Anti-PDL1	−	
	Avelumab	PDL1	Anti-PDL1	−	
	Durvalumab	PDL1	Anti-PDL1	NCT02733042	
	Ipilimumab	CTLA4	Anti-CTLA4	NCT01822509	
	Tremelimumab	CTLA4	Anti-CTLA4	−	
	Pidilizumab	PD1	Anti-PD1	−	[116]
Immunomodulatory Drugs (IMID)	Thalidomide	Cereblon, ikaros, aiolos	Targeting Cereblon, Ikaros, and aiolos with anti-angiogenic, anti-proliferative, and immune-biologic activities	NCT00006226	[117]
	Lenalidomide	Cereblon, ikaros, aiolos	Targeting Cereblon, Ikaros, and aiolos with anti-angiogenic, anti-proliferative, and immune-biologic activities	NCT00632359	[117]
	Pomalidomide	Cereblon, ikaros, aiolos	Targeting Cereblon, Ikaros, and aiolos with anti-angiogenic, anti-proliferative, and immune-biologic activities	−	[117]
Adhesion molecules	BKT140	CXCR4	A small modified peptide CXCR4 antagonist	−	
	AMD3100 (Plerixafor)	CXCR4	A small molecule against CXCR4 that blocks chemotaxis	NCT00694590	[118]
	MDX-1338	CXCR4	An antibody against CXCR4	NCT01120457	
	NOX-A12	CXCL12	Binding and neutralizing CXCL12 instead of binding to CXCR4	NCT01486797	[119]
	Natalizumab	VLA4, α4β7	A humanized mAb that inhibits VLA-4 and the closely related α4β7 integrin	−	[120]
	TBC3486	VLA4	Binding to VLA4 with higher affinity than α4β7 (outbid VCAM-1)	−	
Hypoxia	Echinomycin	HIF1-α	Inhibitor of HIF1-α that blocks hypoxia-dependent drug-resistance	−	[121]

Carfilzomib displayed significant action in ALL cell lines, except P-glycoprotein-positive T-ALL cell lines [95]. In addition, The deletion of the IKZF1 gene was linked to a better response to carfilzomib therapy [112], establishing carfilzomib in combination with existing chemotherapy regimens as an innovative treatment choice for refractory ALL.

### Immunotherapy agents

#### CAR-T cell

Chimeric antigen receptor (CAR)-T cell therapy has been remarkable since it has given rise to exceptionally effective and persistent clinical responses [122]. CARs are synthetically generated receptors that attempt to redirect lymphocytes, usually T cells, so they can identify and destroy cells that express a particular target antigen. Potent anti-tumor responses and robust T-cell activation are the outcomes of CAR binding, which occurs independently of the MHC receptor, to target antigens manufactured on the cell surface [123]. While CAR-T cell immunotherapy can significantly improve therapeutic results, two main side effects must be dealt with: immune effector cell-associated neurotoxicity syndrome (ICANS) and cytokine release syndrome (CRS), both of which can cause mortality and morbidity [124]. The four primary parts of CARs are an extracellular target antigen binding domain, a hinge region, a transmembrane domain, and one or more intracellular signaling domains [125]. The antigen binding domain is the part of the CAR responsible for target antigen selection. Historically, antigen-binding domains were produced from the variable heavy (VH) and light (VL) chains of monoclonal antibodies, which were linked together via a flexible linker to form a single chain variable fragment (scFv). Traditionally, scFvs present in CARs target extracellular surface tumor antigens, resulting in major histocompatibility complex (MHC) independent T cell activation. However, recognition of intracellular tumor-associated antigens using MHC-dependent, T cell receptor (TCR)-mimic CARs has been described [126]. Co-stimulatory and signaling regions comprise the region of intracellular activation. Different CAR-T cell co-stimulatory regions have generated different dynamics. CD28 and 4-1BB are the two primary co-stimulatory areas. Compared to CARs expressing CD28, those expressing 4-1BB exhibited a slower rate of growth but a longer duration of activity [127].

Kymriah, the first FDA-approved CAR T-cell therapy, showed significant efficacy in treating B-ALL, with an 81% overall remission within 3 months [93]. Kymriah targets CD19, a B-cell surface marker, causing a tolerated B-cell aplasia as an anti-tumor effect [128]. Other targets, rather than CD19, are being employed because of tumor antigen loss or CAR T-cell exhaustion. With three distinct groups and various CAR T-cell dosages, the AMELIA trial (NCT03289455) produced over 75% complete responders using CD19 and CD22 as targets. Clinical trials using CAR T-cell treatments are focusing on CD7 (NCT04572308, NCT04033302) or CD5 (NCT04594135) as potential targets for T-ALL treatment [129]. The efficiency of CAR-T cell therapy in CLL is controversial. According to Porter et al., three patients with relapsed or resistant CLL were treated with CD19 CAR T cells, and one patient had a malignant response that lasted longer than ten months [130]. A randomized dose optimization study of CD19 CAR-T cells yielded long-term findings showing a 44% overall response rate and a median overall survival of 64 months [131].

#### Immune Checkpoint Blockade (ICB) therapy

The activation of immune checkpoint pathways is a primary mechanism underpinning tumor immune evasion. Immune checkpoint molecules control the immune system in a physiological setting by inducing and suppressing immune reactions to lessen the immune response when an infection or other threat has been successfully countered. However, similar immune checkpoint interactions may potentially be present in hematological malignancies, and there is rising interest in targeting these to improve anti-tumor immunity [132].

A type of monoclonal antibody-based immunotherapy known as immune checkpoint blockade (ICB) tries to prevent inhibitory receptors (immune checkpoints) expressed on the surface of immune cells from interacting with their ligands. PD-L1, PD-L2, PD-1, and CTLA-4 are the primary targets for these drugs. ICB therapies were developed to improve anti-tumor immune responses, which are primarily regulated by T cells [133].

Immune checkpoint blockade therapies such as PD-1 antibodies such as Pembrolizumab and Nivolumab, anti-PD-L1 antibodies such as Avelumab, Durvalumab, and Atezolizumab, and humanized CTLA-4 antibodies such as Tremelimumab and Ipilimumab have exhibited powerful and lasting anti-tumor action. The PD-1 antibody Pidilizumab (CT-011) showed clinical responses in over 33% of patients with hematological malignancies, including CLL (Table 1) [116].

The existence of tumor neo-antigens, which are associated with a high mutational load, is one of the main factors for successful ICB. However, as compared to highly mutated solid tumors, B-ALL has very low mutational loads, except in cases with mutations affecting DNA mismatch repair genes, making these diseases unfavorable candidates for ICB [134].

#### BiTE

BiTE (bispecific T-cell engager) therapies link endogenous T cells to tumor-expressed antigens, activating the cytotoxic potential of a patient’s T cells to eliminate cancer without genetic alteration of the T cells or the need for ex vivo expansion/manipulation [135, 136]. BiTE molecules can be used as monotherapies and offer enhanced activity in combination with other treatments. Blinatumomab, the first and currently only approved BiTE therapy, targets the CD19 receptor on both normal and malignant B cells and is a highly potent molecule with cytotoxic effects observed at low exposures. In its presence, T cells can perform serial-target lysis, rapidly binding and killing many cells [137]. The efficacy and safety of blinatumomab is established in acute lymphoblastic leukemia (ALL), having received US Food and Drug Administration-accelerated approval in 2014. Since CD19 is broadly and consistently expressed throughout B-cell development, it is an attractive target across all B-cell malignancies.

### Immunomodulatory drugs (IMiD)

Immunomodulatory drugs (IMiDs) include thalidomide and its analogs pomalidomide and lenalidomide. The two first-in-class IMiDs, pomalidomide, and lenalidomide, are synthesized by attaching an amino group to the fourth carbon of thalidomide’s phthaloyl ring [117]. These drugs exhibit pleiotropic actions in hematological malignancies, including anti-angiogenic, anti-proliferative, and immune-biologic activities through direct cytotoxicity against tumor cells and indirect interference with TME cellular components (Table 1) [138].

Thalidomide has also been reported to co-stimulate T cells and trigger NK cells, among other immunomodulatory effects [139]. Preclinical investigations have indicated that IMiD therapy improves T cell co-stimulation and growth by increasing pro-inflammatory cytokines like IL-2, TNF-α, and IFN-γ, and lowering anti-inflammatory cytokines like TGF-β and IL-10 in CLL [140].

Cereblon (CRBN) has been suggested to be the primary direct target for all IMiD therapeutic activity [117]. Ikaros (IKZF1) and Aiolos (IKZF3), two zinc finger-containing transcription factors involved in lymphoid proliferation, are retargeted by IMiDs, leading to their proteasomal destruction [141]. Ikaros and Aiolos at lower concentrations directly inhibit tumor cell proliferation and exhibit anti-neoplastic actions [141].

In CLL cells, lenalidomide may increase p21 expression and cause cell cycle stoppage [142]. Lenalidomide may boost T-cell adherence and migration in CLL by recovering normal Rho-GTPase family (Rho, Rac1, and Cdc42) levels and LFA-1 function [143]. Styczynski et al. discovered that thalidomide had a minor cytotoxic effect in human ALL cell lines and pediatric ALL specimens. However, when combined with prednisolone and cytarabine, thalidomide dramatically improved leukemic cell sensitivity to these drugs in children with ALL [144].

### Targeting adhesion molecules

Several CXCR4 antagonist groups are in clinical trials, including a small modified peptide CXCR4 antagonist (BKT140), a small molecule CXCR4 antagonist (AMD3100, now called Plerixafor), an antibody against CXCR4 (MDX-1338/BMS 93656), and the CXCL12 antagonist NOX- A12 (Table 1) [145]. These drugs are the antagonists of the CXCR4 receptor and prevent CXCL12 binding, reducing pseudo-emperipolesis and chemo-resistance to various drugs [146]. A recent clinical trial combining rituximab (an anti-CD20 antibody) and plerixafor demonstrated increased peripheral blood cell migration, however, only 38% of patients responded [118]. The goal of this investigation was to see if leukemic cells could be recruited from tissues with CXCR4 antagonists and targeted outside of protected tissues. Plerixafor demonstrated a dose-dependent migration of CLL cells from tissues into the blood in this investigation [118]. Other authors have attempted to target this axis by reducing CXCR4 expression with CXCR4 antibodies [147] or histone deacetylase inhibitors [148]. A different approach considered was to target the CXCL12 ligand. NOX-A12, an L-configured RNA oligonucleotide that binds to and blocks CXCL12, has been demonstrated to reduce migration and boost chemosensitivity in CLL B-cells while increasing pseudo-emperipolesis [119]. Furthermore, AMD3100 proved to be capable of inhibiting chemotaxis in primary B-ALL cells and NALM6, as well as potentiating the impact of growth factors and cytokines found in the TME [149].

Given that VLA-4 is a known modulator of chemoresistance in ALL cells, there is growing enthusiasm for enhancing chemosensitivity by using anti-VLA-4 therapies. A humanized monoclonal antibody called natalizumab inhibits VLA-4 and the associated α4β7 integrin, which also binds to VCAM-1, by specifically targeting the α4 integrin subunit [150]. Natalizumab has been demonstrated to drastically diminish stromal adherence in primary B-ALL cells, making them susceptible to chemotherapy and increasing the survival of B-ALL-bearing animals [120] when paired with multi-agent chemotherapy that included dexamethasone, L-asparaginase, and vincristine, natalizumab dramatically extended survival in a xenograft model of B-ALL compared to chemotherapy alone, according to Hsieh et al. Other treatment strategies include peptide or nonpeptide ligands that outbid VCAM-1 for binding to VLA-4. TBC3486 was discovered to increase chemosensitivity in vitro in B-ALL cells co-cultured with BMSCs and in vivo in a xenograft model of B-ALL. It has a noticeably higher affinity for VLA-4 in comparison to α4β7 [151].

### Targeting HIF1-α

Targeting HIF1-α is a potential strategy to block hypoxia-dependent growth, cell proliferation, and MDR [152]. Inhibition of HIF1-α may therefore represent a viable strategy for interfering with disease progression and inducing chemosensitivity in leukemia. Alternatively, Using hypoxia-activated cytotoxic medicines to leverage the hypoxic features of the BM niche could allow for more targeted delivery of treatments to tumor cells [153]. Previous studies have shown that echinomycin inhibits HIF1-α activity in lymphoma and leukemia stem cell models, considerably boosting survival in mouse models and hindering cancer cell progression and proliferation [121]. Therefore, the aim is to develop more specific small molecule inhibitors in ALL to achieve important breakthroughs in reducing angiogenesis.

### Targeting signalling networks

#### BCR inhibitors

BCR inhibitors target different BCR signaling components, including BTK, SYK, and PI3K. BTK inhibitors block effective BCR signaling, which is required for CLL cell growth and TME connections [154]. BTK inhibition induces good response rates and long-term remission in CLL patients, but it does not completely eradicate the illness [155]. The first human BTK inhibitor, ibrutinib, originally known as PCI-32765, selectively and irreversibly binds to cysteine residues in the BTK kinase domain to prevent BTK phosphorylation and its enzymatic activity [156]. Ibrutinib has shown promising clinical results in B-cell leukemias, especially in CLL. Ibrutinib induces apoptosis in CLL cells in the presence of pro-survival CLL elements, as well as suppression of CLL cells viability and growth, leukemic cells migration toward tissue-homing chemokines (CXCL13, CXCL12), and downregulation of BCR-dependent chemokines (CCL3, CCL4) production by CLL cells [157]. Zanubrutinib and acalabrutinib are more specific BTK inhibitors that are currently used effectively in clinics and have shown outcomes similar to ibrutinib while having a reduced incidence of atrial fibrillation [158]. Pirtobrutinib is a third-generation, reversible BTK inhibitor. Initial studies have shown that this drug is selective and effective in B-cell lines in vitro in addition to mouse models [159]. In a study, a total of 62% of patients with CLL (*n* = 139) responded to treatment in a study involving more than 300 B-cell malignant patients. Similarly, pirtobrutinib was equally effective in patients who could not tolerate irreversible BTK inhibitors [160]. Pirtobrutinib’s reversible binding, BTK selection, high effectiveness, attractive pharmacological characteristics, and satisfactory clinical results in patients with wild-type or mutant BTK indicated it might be used to treat any BTK-driven tumor [161]. Selinexor is a selective oral inhibitor of exportin-1 (XPO1)-mediated nuclear transport. The drug’s capacity to target various BCR signaling components independently of BTK kinase function, while maintaining its capacity to inhibit adaptive signaling pathways in ibrutinib-resistant subclones, has been attributed to its ability to overcome ibrutinib-mediated resistance [154].

PI3K inhibitors are another type of BCR inhibitors. Idelalisib, formerly called GS-1101, is a strong, selective, and the first PI3K in clinical trials [162]. Idelalisib causes apoptosis in B-cell lines and primary cells from patients with various B-cell leukemias, such as CLL, and it also inhibits PI3K activity [163]. Patients receiving Idelalisib therapy have an initial redistribution of CLL cells from tissue to blood during the first few weeks of treatment, with transitory lymphocytosis and a decrease in lymph node size. [164]. Duvelisib is a powerful small-molecule oral PI3K inhibitor that targets both the p110δ and p110γ isoforms. It’s an FDA-approved drug for the treatment of relapsed and refractory CLL [165]. Stephens et al. discovered that duvelisib was related to a significant rise in CD8 T-cells. The rise has never been seen in patients receiving idelalisib, indicating that it is a unique effect of PI3K inhibition [166].

SYK inhibitors have an important impact on treating CLL patients. Fostamatinib disodium (FosD), formerly called R788, is a prodrug that quickly transforms in the body into the bioactive molecule R406 [167]. Previous research has revealed that R406 is a selective SYK inhibitor, but it also inhibits other kinases including Lck, Jak, and Flt3 [168]. Entospletinib (GS-9973) is a selective small molecule that disrupts BCR signaling and triggers death in CLL cells. Treatment with entospletinib resulted in lower STAT3 activation and downmodulation of antiapoptotic MCL1 mRNA and protein levels in these settings [169]. However, these results have yet to be verified in vivo (Table 2).

**Table 2:** List of drugs or compounds targeting signaling pathways

Type	Compound/drug	Target	Function	Clinical trial	Reference
BCR inhibitors	Ibrutinib	BTK	BTK phosphorylation by binding selectively and irreversibly to cysteine residues in the BTK kinase domain	NCT02801578	[170]
	Acalabrutinib	BTK	more selective BTK inhibitors than ibrutinib	NCT04930536	[158]
	Zanubrutinib	BTK	more selective BTK inhibitors than ibrutinib	NCT03206918	[158]
	Pirtobrutinib	BTK	Third generation, very effective noncovalent reversible BTK inhibitor	−	[159]
	Selinexor	Exportin-1	target multiple B-cell receptor signaling nodes independently of BTK kinase activity	NCT01607892	[154]
	Idelalisib	PI3K	Selective inhibition of PI3K activation constitutively	NCT02242045	[163]
	Duvelisib	PI3K	PI3K inhibitor that targets both the p110δ and p110γ isoforms with a significant increase in CD8 T-cells	NCT02004522	[165]
	Fostamatinib	SYK	Relatively selective SYK inhibitor	−	[167]
	Entospletinib	SYK	A selective small molecule that disrupts BCR signaling	NCT02983617	[169]
WNT inhibitors	DKK1	LRP5,6	A soluble blocker of WNT-β-catenin signaling by binding to the WNT coreceptor LRP5/6	−	[171]
	XAV939 (Tankyrase inhibitors)	Axin	Member of the PARP family that causes proteasome degradation of axin	−	[67]
	XX-650–23	CBP	CBP inhibitor that improves sensitivity to dasatinib in ALL	−	[172]
	ICG-001	CBP	small-molecule inhibitor of Wnt-β-catenin signaling by binding to the N-terminus of CBP	−	[173]
	Cirmtuzumab	ROR1	An anti ROR-1	NCT02222688	[174]
	CGP049090	LEF1/β-catenin	small molecule inhibitors of LEF1/β-catenin complex	−	
	PKF115-584	LEF1/β-catenin	small molecule inhibitors of LEF1/β-catenin complex	−	
	PF-670462	CK1	CK1 inhibitor	−	
	PF-4800567	CK1	CK1 inhibitor	−	
Notch inhibitors	Demcizumab	DLL4	mAb targeting the Notch ligand DLL4 that disrupts ligand-induced Notch signaling	−	[175]
	Brontictuzumab	Notch1	mAb targeting notch1-NRR region interfering with ADAM 10-dependent processing	−	[176]

#### Targeting WNT signaling

Drugs inhibit WNT signaling in leukemias in a variety of ways. Dickkopf-1 (DKK1), a soluble WNT-β-catenin signaling inhibitor, controls WNT signaling by attaching to the WNT co-receptor LRP5/6. Since the vincristine-resistant BALL-1 cell line (BALL-1/VCR) has considerably greater levels of Wnt3a, Wnt5b, Wnt10a, and LRP5/6, Soluble DKK-1 suppresses the WNT network and improves the responsiveness of BALL-1/VCR to chemotherapy [171].

Relevant targets in the cytoplasmic area of Wnt signaling include CK1, Dvl, Axin, and GSK. The proteasome degrades axin via ubiquitination, which is regulated by tankyrase inhibitors, members of the poly ADP-ribose polymerase (PARP) family of enzymes [177]. In a mouse model, inhibiting the β-catenin pathway with XAV939 (small molecule inhibitor of tankyrases) made B-ALL cells more sensitive to Ara-C therapy and increased the overall response rate [67].

XX-650–23 is a CBP inhibitor, which demonstrated potential efficiency in improving sensitivity to the tyrosine kinase inhibitor (TKI) dasatinib in ALL [172]. ICG-001 is a small-molecule inhibitor of the Wnt-β-catenin network, which breaks the connection between CBP and β-catenin [173]. When paired with regular therapy, ICG-001 was able to eliminate drug-resistant primary B-ALL in vitro and considerably extended the lifespan of a B-ALL mouse model [178].

Because ethacrynic acid (EA)-induced LEF1 transcription activity inhibition increased primary CLL cell apoptosis, targeting the LEF1 transcription function was proposed as a curative approach [179]. LEF1’s DNA binding was interfered with by EA, which also reduced the expression of genes like CCND1 or MYC. CGP049090 and PKF115-584 target the LEF1/β-catenin complex’s transactivation capabilities [157]. Unlike normal B cells, primary CLL cells underwent apoptosis in response to treatment. In a xenograft mouse model, these effects were also observed in vivo [180]. Although clinical trials for selective CK1 inhibitors are yet to start, several drugs, such as PF-670462 and PF-4800567, have been studied in animal examinations and proven to be well-tolerated in vivo [181, 182] and they may be used to treat a variety of Wnt-driven malignancies [183].

Anti-ROR1 monoclonal antibodies can inhibit ROR1 signaling [66, 73]. Data on the ability of antibodies against ROR1 to cause apoptosis in CLL are contradictory [66, 184]. This disagreement can likely be outlined by variations in the epitopes that these inhibitors detect. UC-961 and cirmtuzumab are best-characterized anti-ROR1 antibodies [174]. Chimeric antigen receptor (CAR) T-cells that specifically detect ROR1 have been investigated as a potential alternative strategy that makes use of the particular ROR1 expression in CLL (Table 2) [185].

#### Targeting notch signaling

The Notch network is currently being targeted with small molecules including γ-secretase inhibitors (GSIs) or large molecules like monoclonal antibodies, or mAbs, against several Notch components [186]. GSIs are small substances that serve as Notch inhibitors by suppressing Notch receptor and transmembrane Notch ligand activation. Several investigations have proposed that GSI therapy decreases the amount of active Notch1 protein and reduces Notch target genes in T-ALL cell lines with Notch1-activated alterations, resulting in G1 cell cycle arrest and cell size reduction [76]. Brontictuzumab is a Notch1 inhibitory monoclonal antibody that is capable of blocking the Notch1 network, interfering with ADAM 10-dependent processing but without altering the Notch2 function [176, 187]. Demcizumab targets the Notch ligand DLL4 that disrupts ligand-induced Notch signaling activation in T-ALL cells engrafted in mice, decreasing leukemia cell expansion and boosting apoptosis [175, 188].

## Conclusion

The tumor microenvironment (TME) exerts a pivotal influence on the progression and aggressiveness of lymphoid leukemias, as well as their resistance to conventional therapies. The intricate interplay among various cellular components within the TME, including T-cells, MSCs, and TAMs, results in the secretion of a diverse array of growth factors and cytokines that support the survival, proliferation, and dissemination of leukemic cells. Consequently, there is a compelling need to explore novel strategies aimed at enhancing immune responses. Through the intricate interplay of cytokines, adhesion molecules, and receptors that mediate interactions with tumor cells, these elements collectively establish a suppressive microenvironment conducive to tumor development and progression, thereby contributing to multidrug resistance (MDR).

In response to the challenges posed by MDR, innovative therapeutic modalities have been devised. These novel interventions do not solely target tumor cells but also address TME constituents, such as proteasomes, immune cells, immune checkpoints, adhesion molecules, and signaling pathways. A diverse array of therapeutic agents that target these pathways, including immunotherapy agents, IMiDs, and pathway-specific inhibitors are currently undergoing rigorous evaluation. While the review elucidates numerous potential biomarkers linked to leukemia prognosis and treatment response, the validation of these biomarkers remains a challenge. Furthermore, the identification of novel biomarkers through omics-based methodologies (e.g., genomics, transcriptomics, proteomics) has the potential to refine patient stratification and predict treatment outcomes more accurately. Ongoing investigations into drugs targeting these pathways are crucial, necessitating further research efforts to optimize existing therapies and develop innovative pharmacological agents that can precisely and effectively target these critical pathways. Ultimately, such endeavors hold promise for enhancing treatment outcomes in patients afflicted with lymphoid leukemias.

## Data Availability

No datasets were generated or analysed during the current study.

## References

[1] Arruga F, Gyau BB, Iannello A, Vitale N, Vaisitti T, Deaglio S (2020) Immune response dysfunction in chronic lymphocytic leukemia: dissecting molecular mechanisms and microenvironmental conditions. Int J Mol Sci 21(5) 10.3390/ijms21051825 PMC7084946 32155826

[2] Baghban R, Roshangar L, Jahanban-Esfahlan R, Seidi K, Ebrahimi-Kalan A, Jaymand M et al (2020) Tumor microenvironment complexity and therapeutic implications at a glance. Cell Commun Signal 18(1):59 32264958 10.1186/s12964-020-0530-4 PMC7140346

[3] Cariello M, Squilla A, Piacente M, Venutolo G, Fasano A (2022) Drug resistance: the role of exosomal mirna in the microenvironment of hematopoietic tumors. Molecules 28(1) 10.3390/molecules28010116 PMC9821808 36615316

[4] Xiao Y, Yu D (2021) Tumor microenvironment as a therapeutic target in cancer. Pharmacol Ther 221:107753 33259885 10.1016/j.pharmthera.2020.107753 PMC8084948

[5] Namee NM, O’Driscoll L (2018) Extracellular vesicles and anti-cancer drug resistance. Biochim Biophys acta Rev cancer 1870(2):123–136 30003999 10.1016/j.bbcan.2018.07.003

[6] Tran TH, Hunger SP (2022) The genomic landscape of pediatric acute lymphoblastic leukemia and precision medicine opportunities. Semin Cancer Biol 84:144–152 33197607 10.1016/j.semcancer.2020.10.013

[7] Simioni C, Bergamini F, Ferioli M, Rimondi E, Caruso L, Neri LM (2020) New biomarkers and therapeutic strategies in acute lymphoblastic leukemias: Recent advances. Hematol Oncol 38(1):22–33 31487068 10.1002/hon.2678

[8] Scarfò L, Ferreri AJM, Ghia P (2016) Chronic lymphocytic leukaemia. Crit Rev Oncol Hematol 104:169–182 27370174 10.1016/j.critrevonc.2016.06.003

[9] Kitada S, Andersen J, Akar S, Zapata JM, Takayama S, Krajewski S et al (1998) Expression of apoptosis-regulating proteins in chronic lymphocytic leukemia: correlations with in vitro and in vivo chemoresponses. Blood 91(9):3379–3389 9558396

[10] Hallek M (2019) Chronic lymphocytic leukemia: 2020 update on diagnosis, risk stratification and treatment. Am J Hematol 94(11):1266–1287 31364186 10.1002/ajh.25595

[11] Landau DA, Carter SL, Stojanov P, McKenna A, Stevenson K, Lawrence MS et al (2013) Evolution and impact of subclonal mutations in chronic lymphocytic leukemia. Cell 152(4):714–726 23415222 10.1016/j.cell.2013.01.019 PMC3575604

[12] Schreiber RD, Old LJ, Smyth MJ (2011) Cancer immunoediting: integrating immunity’s roles in cancer suppression and promotion. Science 331(6024):1565–1570 21436444 10.1126/science.1203486

[13] Caplan AI (1991) Mesenchymal stem cells. J Orthop Res Off Publ Orthop Res Soc 9(5):641–650 10.1002/jor.1100090504 1870029

[14] Lindner U, Kramer J, Rohwedel J, Schlenke P (2010) Mesenchymal stem or stromal cells: toward a better understanding of their biology? Transfus Med hemotherapy Off Organ der Dtsch Gesellschaft fur Transfusionsmedizin und Immunhamatologie 37(2):75–83 10.1159/000290897 PMC2914415 20737049

[15] Pontikoglou C, Kastrinaki MC, Klaus M, Kalpadakis C, Katonis P, Alpantaki K et al (2013) Study of the quantitative, functional, cytogenetic, and immunoregulatory properties of bone marrow mesenchymal stem cells in patients with B-cell chronic lymphocytic leukemia. Stem Cells Dev 22(9):1329–1341 23249221 10.1089/scd.2012.0255 PMC3629855

[16] Swiergiel AH, Dunn AJ (1999S) The roles of IL-1, IL-6, and TNFalpha in the feeding responses to endotoxin and influenza virus infection in mice. Brain Behav Immun 13(3):252–265 10469526 10.1006/brbi.1999.0565

[17] Beneforti L, Dander E, Bresolin S, Bueno C, Acunzo D, Bertagna M et al (2020) Pro-inflammatory cytokines favor the emergence of ETV6-RUNX1-positive pre-leukemic cells in a model of mesenchymal niche. Br J Haematol 190(2):262–273 32118299 10.1111/bjh.16523

[18] Zelent A, Greaves M, Enver T (2004) Role of the TEL-AML1 fusion gene in the molecular pathogenesis of childhood acute lymphoblastic leukaemia. Oncogene 23(24):4275–4283 15156184 10.1038/sj.onc.1207672

[19] Conforti A, Biagini S, Del Bufalo F, Sirleto P, Angioni A, Starc N et al (2013) Biological, functional and genetic characterization of bone marrow-derived mesenchymal stromal cells from pediatric patients affected by acute lymphoblastic leukemia. PLoS ONE 8(11):e76989 24244271 10.1371/journal.pone.0076989 PMC3820675

[20] Vicente López Á, Vázquez García MN, Melen GJ, Entrena Martínez A, Cubillo Moreno I, García-Castro J et al (2014) Mesenchymal stromal cells derived from the bone marrow of acute lymphoblastic leukemia patients show altered BMP4 production: correlations with the course of disease. PLoS ONE 9(1):e84496 24400095 10.1371/journal.pone.0084496 PMC3882230

[21] Valencia J, M Fernández-Sevilla L, Fraile-Ramos A, Sacedón R, Jiménez E, Vicente A et al (2019) Acute lymphoblastic leukaemia cells impair dendritic cell and macrophage differentiation: role of BMP4. Cells. 28(7) 10.3390/cells8070722 PMC6679123 31337120

[22] Balandrán JC, Purizaca J, Enciso J, Dozal D, Sandoval A, Jiménez-Hernández E et al (2017) Pro-inflammatory-related loss of CXCL12 niche promotes acute lymphoblastic leukemic progression at the expense of normal lymphopoiesis. Front Immunol 7 10.3389/fimmu.2016.00666 PMC5216624 28111575

[23] Heinig K, Gätjen M, Grau M, Stache V, Anagnostopoulos I, Gerlach K et al (2014) Access to follicular dendritic cells is a pivotal step in murine chronic lymphocytic leukemia B-cell activation and proliferation. Cancer Discov 4(12):1448–1465 25252690 10.1158/2159-8290.CD-14-0096

[24] Chilosi M, Pizzolo G, Caligaris-Cappio F, Ambrosetti A, Vinante F, Morittu L et al (1985) Immunohistochemical demonstration of follicular dendritic cells in bone marrow involvement of B-cell chronic lymphocytic leukemia. Cancer 56(2):328–332 3891066 10.1002/1097-0142(19850715)56:2<328::aid-cncr2820560221>3.0.co;2-q

[25] Allen CDC, Cyster JG (2008) Follicular dendritic cell networks of primary follicles and germinal centers: phenotype and function. Semin Immunol 20(1):14–25 18261920 10.1016/j.smim.2007.12.001 PMC2366796

[26] Fang Y, Xu C, Fu YX, Holers VM, Molina H (1998) Expression of complement receptors 1 and 2 on follicular dendritic cells is necessary for the generation of a strong antigen-specific IgG response. J Immunol 160(11):5273–5279 9605124

[27] Dubois N, Crompot E, Meuleman N, Bron D, Lagneaux L, Stamatopoulos B (2020) Importance of crosstalk between chronic lymphocytic leukemia cells and the stromal microenvironment: direct contact, soluble factors, and extracellular vesicles. Front Oncol 10:1422 32974152 10.3389/fonc.2020.01422 PMC7466743

[28] Murray PJ, Allen JE, Biswas SK, Fisher EA, Gilroy DW, Goerdt S et al (2014) Macrophage activation and polarization: nomenclature and experimental guidelines. Immunity 41(1):14–20 25035950 10.1016/j.immuni.2014.06.008 PMC4123412

[29] Boutilier AJ, Elsawa SF (2021) Macrophage polarization states in the tumor microenvironment. Int J Mol Sci 22(13) 10.3390/ijms22136995 PMC8268869 34209703

[30] Mosser DM, Edwards JP (2008) Exploring the full spectrum of macrophage activation. Nat Rev Immunol 8(12):958–969 19029990 10.1038/nri2448 PMC2724991

[31] Zhu S, Yi M, Wu Y, Dong B, Wu K (2021) Roles of tumor-associated macrophages in tumor progression: implications on therapeutic strategies. Exp Hematol Oncol 10(1):60 34965886 10.1186/s40164-021-00252-z PMC8715617

[32] Martinez FO, Sica A, Mantovani A, Locati M (2008) Macrophage activation and polarization. Front Biosci 13:453–461 17981560 10.2741/2692

[33] Chen SY, Yang X, Feng WL, Liao JF, Wang LN, Feng L et al (2015) Organ-specific microenvironment modifies diverse functional and phenotypic characteristics of leukemia-associated macrophages in mouse T cell acute lymphoblastic leukemia. J Immunol 194(6):2919–2929 25662994 10.4049/jimmunol.1400451

[34] Li C, Xu X, Wei S, Jiang P, Xue L, Wang J (2021) Tumor-associated macrophages: potential therapeutic strategies and future prospects in cancer. J Immunother Cancer. 9(1) 10.1136/jitc-2020-001341 PMC8728363 33504575

[35] Filip AA, Ciseł B, Koczkodaj D, Wąsik-Szczepanek E, Piersiak T, Dmoszyńska A (2013) Circulating microenvironment of CLL: are nurse-like cells related to tumor-associated macrophages? Blood Cells Mol Dis 50(4):263–270 23313631 10.1016/j.bcmd.2012.12.003

[36] Burger JA, Tsukada N, Burger M, Zvaifler NJ, Dell’Aquila M, Kipps TJ (2000) Blood-derived nurse-like cells protect chronic lymphocytic leukemia B cells from spontaneous apoptosis through stromal cell-derived factor-1. Blood 96(8):2655–2663 11023495

[37] Chao MP, Alizadeh AA, Tang C, Jan M, Weissman-Tsukamoto R, Zhao F et al (2011) Therapeutic antibody targeting of CD47 eliminates human acute lymphoblastic leukemia. Cancer Res 71(4):1374–1384 21177380 10.1158/0008-5472.CAN-10-2238 PMC3041855

[38] Xiao A, Akilov OE (2022) Targeting the CD47-SIRPα axis: present therapies and the future for cutaneous T-cell lymphoma. Cells 11(22) 10.3390/cells11223591 PMC9688096 36429020

[39] Okazawa H, Motegi S ichiro, Ohyama N, Ohnishi H, Tomizawa T, Kaneko Y et al (2005) Negative regulation of phagocytosis in macrophages by the CD47-SHPS-1 system. J Immunol. 174(4):2004–2011 10.4049/jimmunol.174.4.2004 15699129

[40] Olcucuoglu E, Sirin ME, Aydog G, Gazel E, Tastemur S, Odabas O (2017) Relationship between immunohistochemical staining extent of CD47 and histopathologic features of bladder tumor. Cent Eur J Urol 70(4):349–355 10.5173/ceju.2017.1357 PMC5791396 29410884

[41] Liu Z, Zhou Z, Dang Q, Xu H, Lv J, Li H et al (2022) Immunosuppression in tumor immune microenvironment and its optimization from CAR-T cell therapy. Theranostics 12(14):6273–6290 36168626 10.7150/thno.76854 PMC9475465

[42] Rudd CE, Taylor A, Schneider H (2009) CD28 and CTLA-4 coreceptor expression and signal transduction. Immunol Rev 229(1):12–26 19426212 10.1111/j.1600-065X.2009.00770.x PMC4186963

[43] Wing K, Onishi Y, Prieto-Martin P, Yamaguchi T, Miyara M, Fehervari Z et al (2008) CTLA-4 control over Foxp3+ regulatory T cell function. Science 322(5899):271–275 18845758 10.1126/science.1160062

[44] Freeman GJ, Long AJ, Iwai Y, Bourque K, Chernova T, Nishimura H et al (2000) Engagement of the PD-1 immunoinhibitory receptor by a novel B7 family member leads to negative regulation of lymphocyte activation. J Exp Med 192(7):1027–1034 11015443 10.1084/jem.192.7.1027 PMC2193311

[45] Jiang Y, Chen M, Nie H, Yuan Y (2019) PD-1 and PD-L1 in cancer immunotherapy: clinical implications and future considerations. Hum Vaccin Immunother 15(5):1111–1122 30888929 10.1080/21645515.2019.1571892 PMC6605868

[46] Ramsay AG, Clear AJ, Fatah R, Gribben JG (2012) Multiple inhibitory ligands induce impaired T-cell immunologic synapse function in chronic lymphocytic leukemia that can be blocked with lenalidomide: establishing a reversible immune evasion mechanism in human cancer. Blood 120(7):1412–1421 22547582 10.1182/blood-2012-02-411678 PMC3423779

[47] Schwartz RH (1992) Costimulation of T lymphocytes: the role of CD28, CTLA-4, and B7/BB1 in interleukin-2 production and immunotherapy. Cell 71(7):1065–1068 1335362 10.1016/s0092-8674(05)80055-8

[48] Ohue Y, Nishikawa H (2019) Regulatory T (Treg) cells in cancer: Can Treg cells be a new therapeutic target? Cancer Sci 110(7):2080–2089 31102428 10.1111/cas.14069 PMC6609813

[49] Ilcus C, Bagacean C, Tempescul A, Popescu C, Parvu A, Cenariu M et al (2017) Immune checkpoint blockade: the role of PD-1-PD-L axis in lymphoid malignancies. Onco Targets Ther 10:2349–2363 28496333 10.2147/OTT.S133385 PMC5417656

[50] Yan H, Dong M, Liu X, Shen Q, He D, Huang X et al (2019) Multiple myeloma cell-derived IL-32γ increases the immunosuppressive function of macrophages by promoting indoleamine 2,3-dioxygenase (IDO) expression. Cancer Lett 446:38–48 30660652 10.1016/j.canlet.2019.01.012

[51] Gupta YH, Khanom A, Acton SE (2022) Control of dendritic cell function within the tumour microenvironment. Front Immunol 13:733800 35355992 10.3389/fimmu.2022.733800 PMC8960065

[52] Niiro H, Clark EA (2002) Regulation of B-cell fate by antigen-receptor signals. Nat Rev Immunol 2(12):945–956 12461567 10.1038/nri955

[53] Davis RE, Ngo VN, Lenz G, Tolar P, Young RM, Romesser PB et al (2010) Chronic active B-cell-receptor signalling in diffuse large B-cell lymphoma. Nature 463(7277):88–92 20054396 10.1038/nature08638 PMC2845535

[54] Shaffer AL 3rd, Young RM, Staudt LM (2012) Pathogenesis of human B cell lymphomas. Annu Rev Immunol 30:565–610 22224767 10.1146/annurev-immunol-020711-075027 PMC7478144

[55] Woyach JA, Johnson AJ, Byrd JC (2012) The B-cell receptor signaling pathway as a therapeutic target in CLL. Blood 120(6):1175–1184 22715122 10.1182/blood-2012-02-362624 PMC3418714

[56] Genevier HC, Hinshelwood S, Gaspar HB, Rigley KP, Brown D, Saeland S et al (1994) Expression of Bruton’s tyrosine kinase protein within the B cell lineage. Eur J Immunol 24(12):3100–3105 7805739 10.1002/eji.1830241228

[57] Petro JB, Rahman SM, Ballard DW, Khan WN (2000) Bruton’s tyrosine kinase is required for activation of IkappaB kinase and nuclear factor kappaB in response to B cell receptor engagement. J Exp Med 191(10):1745–1754 10811867 10.1084/jem.191.10.1745 PMC2193161

[58] Turner M, Mee PJ, Costello PS, Williams O, Price AA, Duddy LP et al (1995) Perinatal lethality and blocked B-cell development in mice lacking the tyrosine kinase Syk. Nature 378(6554):298–302 7477352 10.1038/378298a0

[59] Srinivasan L, Sasaki Y, Calado DP, Zhang B, Paik JH, DePinho RA et al (2009) PI3 kinase signals BCR-dependent mature B cell survival. Cell 139(3):573–586 19879843 10.1016/j.cell.2009.08.041 PMC2787092

[60] Okkenhaug K, Vanhaesebroeck B (2003) PI3K in lymphocyte development, differentiation and activation. Nat Rev Immunol 3(4):317–330 12669022 10.1038/nri1056

[61] Peiffer L, Poll-Wolbeck SJ, Flamme H, Gehrke I, Hallek M, Kreuzer KA (2014) Trichostatin A effectively induces apoptosis in chronic lymphocytic leukemia cells via inhibition of Wnt signaling and histone deacetylation. J Cancer Res Clin Oncol 140(8):1283–1293 24793644 10.1007/s00432-014-1689-0 PMC11824153

[62] Clevers H, Nusse R (2012) Wnt/β-catenin signaling and disease. Cell 149(6):1192–1205 22682243 10.1016/j.cell.2012.05.012

[63] Khan NI, Bradstock KF, Bendall LJ (2007) Activation of Wnt/beta-catenin pathway mediates growth and survival in B-cell progenitor acute lymphoblastic leukaemia. Br J Haematol 138(3):338–348 17614820 10.1111/j.1365-2141.2007.06667.x

[64] Guo X, Zhang R, Liu J, Li M, Song C, Dovat S et al (2015) Characterization of LEF1 High Expression and Novel Mutations in Adult Acute Lymphoblastic Leukemia. PLoS ONE 10(5):1–13 10.1371/journal.pone.0125429 PMC4420493 25942645

[65] Meyer LK, Hermiston ML (2019) The bone marrow microenvironment as a mediator of chemoresistance in acute lymphoblastic leukemia. Cancer drug Resist (Alhambra, Calif) 2(4):1164–1177 10.20517/cdr.2019.63 PMC9019215 35582273

[66] Yang J, Baskar S, Kwong KY, Kennedy MG, Wiestner A, Rader C (2011) Therapeutic potential and challenges of targeting receptor tyrosine kinase ROR1 with monoclonal antibodies in B-cell malignancies. PLoS ONE 6(6):e21018 21698301 10.1371/journal.pone.0021018 PMC3115963

[67] Kotašková J, Pavlová Š, Greif I, Stehlíková O, Plevová K, Janovská P et al (2016) ROR1-based immunomagnetic protocol allows efficient separation of CLL and healthy B cells. British J Haematol England 175:39–42 10.1111/bjh.13848 26567475

[68] Karvonen H, Perttilä R, Niininen W, Hautanen V, Barker H, Murumägi A et al (2019) Wnt5a and ROR1 activate non-canonical Wnt signaling via RhoA in TCF3-PBX1 acute lymphoblastic leukemia and highlight new treatment strategies via Bcl-2 co-targeting. Oncogene 38(17):3288–3300 30631148 10.1038/s41388-018-0670-9

[69] Behrens J, von Kries JP, Kühl M, Bruhn L, Wedlich D, Grosschedl R et al (1996) Functional interaction of beta-catenin with the transcription factor LEF-1. Nature 382(6592):638–642 8757136 10.1038/382638a0

[70] Huber O, Korn R, McLaughlin J, Ohsugi M, Herrmann BG, Kemler R (1996) Nuclear localization of beta-catenin by interaction with transcription factor LEF-1. Mech Dev 59(1):3–10 8892228 10.1016/0925-4773(96)00597-7

[71] Reya T, O’Riordan M, Okamura R, Devaney E, Willert K, Nusse R et al (2000) Wnt signaling regulates B lymphocyte proliferation through a LEF-1 dependent mechanism. Immunity 13(1):15–24 10933391 10.1016/s1074-7613(00)00004-2

[72] Wang L, Shalek AK, Lawrence M, Ding R, Gaublomme JT, Pochet N et al (2014) Somatic mutation as a mechanism of Wnt/β-catenin pathway activation in CLL. Blood 124(7):1089–1098 24778153 10.1182/blood-2014-01-552067 PMC4133483

[73] Kaucká M, Plevová K, Pavlová Š, Janovská P, Mishra A, Verner J et al (2013) The planar cell polarity pathway drives pathogenesis of chronic lymphocytic leukemia by the regulation of B-lymphocyte migration. Cancer Res 73(5):1491–1501 23338609 10.1158/0008-5472.CAN-12-1752

[74] Kaucká M, Petersen J, Janovská P, Radaszkiewicz T, Smyčková L, Daulat AM et al (2015) Asymmetry of VANGL2 in migrating lymphocytes as a tool to monitor activity of the mammalian WNT/planar cell polarity pathway. Cell Commun Signal 13:2 25627785 10.1186/s12964-014-0079-1 PMC4314808

[75] Bellavia D, Palermo R, Felli MP, Screpanti I, Checquolo S (2018) Notch signaling as a therapeutic target for acute lymphoblastic leukemia. Expert Opin Ther Targets 22(4):331–342 29527929 10.1080/14728222.2018.1451840

[76] Weng AP, Ferrando AA, Lee W, Morris JP 4th, Silverman LB, Sanchez-Irizarry C et al (2004) Activating mutations of NOTCH1 in human T cell acute lymphoblastic leukemia. Science 306(5694):269–271 15472075 10.1126/science.1102160

[77] Takebe N, Miele L, Harris PJ, Jeong W, Bando H, Kahn M et al (2015) Targeting Notch, Hedgehog, and Wnt pathways in cancer stem cells: clinical update. Nat Rev Clin Oncol 12(8):445–464 25850553 10.1038/nrclinonc.2015.61 PMC4520755

[78] Wang W, Zimmerman G, Huang X, Yu S, Myers J, Wang Y et al (2016) Aberrant notch signaling in the bone marrow microenvironment of acute lymphoid leukemia suppresses osteoblast-mediated support of hematopoietic niche function. Cancer Res 76(6):1641–1652 26801976 10.1158/0008-5472.CAN-15-2092 PMC4794354

[79] Rosati E, Sabatini R, Rampino G, Tabilio A, Di Ianni M, Fettucciari K et al (2009) Constitutively activated notch signaling is involved in survival and apoptosis resistance of B-CLL cells. Blood 113(4):856–865 18796623 10.1182/blood-2008-02-139725

[80] Ntoufa S, Vilia MG, Stamatopoulos K, Ghia P, Muzio M (2016) Toll-like receptors signaling: a complex network for NF-κB activation in B-cell lymphoid malignancies. Semin Cancer Biol 39:15–25 27402288 10.1016/j.semcancer.2016.07.001

[81] de Lourdes PA, Amarante MK, Guembarovski RL, de Oliveira CEC, Watanabe MAE (2015) CXCL12/CXCR4 axis in the pathogenesis of acute lymphoblastic leukemia (ALL): a possible therapeutic target. Cell Mol Life Sci 72(9):1715–1723 25572297 10.1007/s00018-014-1830-x PMC11113340

[82] Naci D, Aoudjit F (2014) Alpha2beta1 integrin promotes T cell survival and migration through the concomitant activation of ERK/Mcl-1 and p38 MAPK pathways. Cell Signal 26(9):2008–2015 24880062 10.1016/j.cellsig.2014.05.016

[83] Möhle R, Failenschmid C, Bautz F, Kanz L (1999) Overexpression of the chemokine receptor CXCR4 in B cell chronic lymphocytic leukemia is associated with increased functional response to stromal cell-derived factor-1 (SDF-1). Leukemia 13(12):1954–1959 10602415 10.1038/sj.leu.2401602

[84] Calissano C, Damle RN, Hayes G, Murphy EJ, Hellerstein MK, Moreno C et al (2009) In vivo intraclonal and interclonal kinetic heterogeneity in B-cell chronic lymphocytic leukemia. Blood 114(23):4832–4842 19789386 10.1182/blood-2009-05-219634 PMC2925477

[85] Butcher EC, Picker LJ (1996) Lymphocyte homing and homeostasis. Science 272(5258):60–66 8600538 10.1126/science.272.5258.60

[86] Shalapour S, Hof J, Kirschner-Schwabe R, Bastian L, Eckert C, Prada J et al (2011) High VLA-4 expression is associated with adverse outcome and distinct gene expression changes in childhood B-cell precursor acute lymphoblastic leukemia at first relapse. Haematologica 96(11):1627–1635 21828124 10.3324/haematol.2011.047993 PMC3208680

[87] Burger JA, Burger M, Kipps TJ (1999) Chronic lymphocytic leukemia B cells express functional CXCR4 chemokine receptors that mediate spontaneous migration beneath bone marrow stromal cells. Blood 94(11):3658–3667 10572077

[88] Burger JA, Zvaifler NJ, Tsukada N, Firestein GS, Kipps TJ (2001) Fibroblast-like synoviocytes support B-cell pseudoemperipolesis via a stromal cell-derived factor-1- and CD106 (VCAM-1)-dependent mechanism. J Clin Invest 107(3):305–315 11160154 10.1172/JCI11092 PMC199194

[89] Jacamo R, Chen Y, Wang Z, Ma W, Zhang M, Spaeth EL et al (2014) Reciprocal leukemia-stroma VCAM-1/VLA-4-dependent activation of NF-κB mediates chemoresistance. Blood 123(17):2691–2702 24599548 10.1182/blood-2013-06-511527 PMC3999754

[90] Mudry RE, Fortney JE, York T, Hall BM, Gibson LF (2000) Stromal cells regulate survival of B-lineage leukemic cells during chemotherapy. Blood 96(5):1926–1932 10961896

[91] Dander E, Palmi C, D’Amico G, Cazzaniga G (2021) The bone marrow niche in B-cell acute lymphoblastic leukemia: the role of microenvironment from pre-leukemia to overt leukemia. Int J Mol Sci 22(9) 10.3390/ijms22094426 PMC8122951 33922612

[92] Bürkle A, Niedermeier M, Schmitt-Gräff A, Wierda WG, Keating MJ, Burger JA (2007) Overexpression of the CXCR5 chemokine receptor, and its ligand, CXCL13 in B-cell chronic lymphocytic leukemia. Blood 110(9):3316–3325 17652619 10.1182/blood-2007-05-089409

[93] Maude SL, Laetsch TW, Buechner J, Rives S, Boyer M, Bittencourt H et al (2018) Tisagenlecleucel in children and young adults with B-Cell lymphoblastic leukemia. N Engl J Med 378(5):439–448 29385370 10.1056/NEJMoa1709866 PMC5996391

[94] Jing X, Yang F, Shao C, Wei K, Xie M, Shen H et al (2019) Role of hypoxia in cancer therapy by regulating the tumor microenvironment. Mol Cancer 18(1):157 31711497 10.1186/s12943-019-1089-9 PMC6844052

[95] Méndez-Ferrer S, Bonnet D, Steensma DP, Hasserjian RP, Ghobrial IM, Gribben JG et al (2020) Bone marrow niches in haematological malignancies. Nat Rev Cancer 20(5):285–298 32112045 10.1038/s41568-020-0245-2 PMC9912977

[96] Wellmann S, Guschmann M, Griethe W, Eckert C, von Stackelberg A, Lottaz C et al (2004) Activation of the HIF pathway in childhood ALL, prognostic implications of VEGF. Leukemia 18(5):926–933 15014526 10.1038/sj.leu.2403332

[97] Thiery JP, Acloque H, Huang RYJ, Nieto MA (2009) Epithelial-mesenchymal transitions in development and disease. Cell 139(5):871–890 19945376 10.1016/j.cell.2009.11.007

[98] Steinbichler TB, Dudás J, Skvortsov S, Ganswindt U, Riechelmann H, Skvortsova II (2019) Therapy resistance mediated by exosomes. Mol Cancer 18(1):58 30925921 10.1186/s12943-019-0970-x PMC6441190

[99] Kalluri R, LeBleu VS (2020) The biology, function, and biomedical applications of exosomes. Science 367(6478) 10.1126/science.aau6977 PMC7717626 32029601

[100] He G, Peng X, Wei S, Yang S, Li X, Huang M et al (2022) Exosomes in the hypoxic TME: from release, uptake and biofunctions to clinical applications. Mol Cancer 21(1):19. 10.1186/s12943-021-01440-5 10.1186/s12943-021-01440-5 PMC8762953 35039054

[101] Kalluri R (2016) The biology and function of exosomes in cancer. J Clin Invest 126(4):1208–1215 27035812 10.1172/JCI81135 PMC4811149

[102] Guo QR, Wang H, da Yan Y, Liu Y, Su CY, Chen HB et al (2020) The role of exosomal microRNA in cancer drug resistance. Front Oncol 10:472 32318350 10.3389/fonc.2020.00472 PMC7154138

[103] Guo C, Liu J, Zhou Q, Song J, Zhang Z, Li Z et al (2020) Exosomal noncoding RNAs and tumor drug resistance. Cancer Res 80(20):4307–4313 32641408 10.1158/0008-5472.CAN-20-0032

[104] Zamani A, Fattahi Dolatabadi N, Houshmand M, Nabavizadeh N (2021) miR-324-3p and miR-508-5p expression levels could serve as potential diagnostic and multidrug-resistant biomarkers in childhood acute lymphoblastic leukemia. Leuk Res 109:106643 34147937 10.1016/j.leukres.2021.106643

[105] Jaiswal R, Luk F, Gong J, Mathys JM, Grau GER, Bebawy M (2012) Microparticle conferred microRNA profiles–implications in the transfer and dominance of cancer traits. Mol Cancer 11:37 22682234 10.1186/1476-4598-11-37 PMC3499176

[106] Bouvy C, Wannez A, Laloy J, Chatelain C, Dogné JM (2017) Transfer of multidrug resistance among acute myeloid leukemia cells via extracellular vesicles and their microRNA cargo. Leuk Res 62:70–76 28987820 10.1016/j.leukres.2017.09.014

[107] Bestor TH (1988) Cloning of a mammalian DNA methyltransferase. Gene 74(1):9–12 3248734 10.1016/0378-1119(88)90238-7

[108] Rahmani T, Azad M, Chahardouli B, Nasiri H, Vatanmakanian M, Kaviani S (2017) atterns of DNMT1 promoter methylation in patients with acute lymphoblastic leukemia. Int J Hematol Stem Cell Res 11(3):172–177 PMC5625466 28989582

[109] Kn H, Bassal S, Tikellis C, El-Osta A (2004) Expression analysis of the epigenetic methyltransferases and methyl-CpG binding protein families in the normal B-cell and B-cell chronic lymphocytic leukemia (CLL). Cancer Biol Ther 3(10):989–994 15467427 10.4161/cbt.3.10.1137

[110] Jiang H, Ou Z, He Y, Yu M, Wu S, Li G et al (2020) DNA methylation markers in the diagnosis and prognosis of common leukemias. Signal Transduct Target Ther 5(1):3 32296024 10.1038/s41392-019-0090-5 PMC6959291

[111] Crawford LJ, Walker B, Irvine AE (2011) Proteasome inhibitors in cancer therapy. J Cell Commun Signal 5(2):101–110 21484190 10.1007/s12079-011-0121-7 PMC3088792

[112] Takahashi K, Inukai T, Imamura T, Yano M, Tomoyasu C, Lucas DM et al (2017) Anti-leukemic activity of bortezomib and carfilzomib on B-cell precursor aLL cell lines. PLoS ONE 12(12):e0188680 29236701 10.1371/journal.pone.0188680 PMC5728482

[113] Gandolfi S, Laubach JP, Hideshima T, Chauhan D, Anderson KC, Richardson PG (2017) The proteasome and proteasome inhibitors in multiple myeloma. Cancer Metastasis Rev 36(4):561–584 29196868 10.1007/s10555-017-9707-8

[114] Tan CRC, Abdul-Majeed S, Cael B, Barta SK (2019) Clinical pharmacokinetics and pharmacodynamics of bortezomib. Clin Pharmacokinet 58(2):157–168 29802543 10.1007/s40262-018-0679-9

[115] August KJ, Guest EM, Lewing K, Hays JA, Gamis AS (2020) Treatment of children with relapsed and refractory acute lymphoblastic leukemia with mitoxantrone, vincristine, pegaspargase, dexamethasone, and bortezomib. Pediatr Blood Cancer 67(3):e28062 31724803 10.1002/pbc.28062

[116] Berger R, Rotem-Yehudar R, Slama G, Landes S, Kneller A, Leiba M et al (2008) Phase I safety and pharmacokinetic study of CT-011, a humanized antibody interacting with PD-1, in patients with advanced hematologic malignancies. Clin Cancer Res An Off J Am Assoc Cancer Res 14(10):3044–3051 10.1158/1078-0432.CCR-07-4079 18483370

[117] Ito T, Handa H (2020) Molecular mechanisms of thalidomide and its derivatives. Proc Jpn Acad Ser B Phys Biol Sci 96(6):189–203 32522938 10.2183/pjab.96.016 PMC7298168

[118] Andritsos LA, Byrd JC, Cheverton P, Wu J, Sivina M, Kipps TJ et al (2019) A multicenter phase 1 study of plerixafor and rituximab in patients with chronic lymphocytic leukemia. Leuk Lymphoma 60(14):3461–3469 31352850 10.1080/10428194.2019.1643463

[119] Hoellenriegel J, Zboralski D, Maasch C, Rosin NY, Wierda WG, Keating MJ et al (2014) The Spiegelmer NOX-A12, a novel CXCL12 inhibitor, interferes with chronic lymphocytic leukemia cell motility and causes chemosensitization. Blood 123(7):1032–1039 24277076 10.1182/blood-2013-03-493924 PMC4123413

[120] Hsieh YT, Gang EJ, Geng H, Park E, Huantes S, Chudziak D et al (2013) Integrin alpha4 blockade sensitizes drug resistant pre-B acute lymphoblastic leukemia to chemotherapy. Blood 121(10):1814–1818 23319569 10.1182/blood-2012-01-406272 PMC3591800

[121] Wang Y, Liu Y, Malek SN, Zheng P, Liu Y (2011) Targeting HIF1α eliminates cancer stem cells in hematological malignancies. Cell Stem Cell 8(4):399–411 21474104 10.1016/j.stem.2011.02.006 PMC3084595

[122] June CH, O’Connor RS, Kawalekar OU, Ghassemi S, Milone MC (2018) CAR T cell immunotherapy for human cancer. Science 359(6382):1361–1365 29567707 10.1126/science.aar6711

[123] Sadelain M, Brentjens R, Rivière I (2013) The basic principles of chimeric antigen receptor design. Cancer Discov 3(4):388–398 23550147 10.1158/2159-8290.CD-12-0548 PMC3667586

[124] Han X, Wang Y, Wei J, Han W (2019) Multi-antigen-targeted chimeric antigen receptor T cells for cancer therapy. J Hematol Oncol 12(1):128 31783889 10.1186/s13045-019-0813-7 PMC6884912

[125] Sterner RC, Sterner RM (2021) CAR-T cell therapy: current limitations and potential strategies. Blood Cancer J 11(4):69 33824268 10.1038/s41408-021-00459-7 PMC8024391

[126] Whitlow M, Bell BA, Feng SL, Filpula D, Hardman KD, Hubert SL et al (1993) An improved linker for single-chain Fv with reduced aggregation and enhanced proteolytic stability. Protein Eng 6(8):989–995 8309948 10.1093/protein/6.8.989

[127] Han D, Xu Z, Zhuang Y, Ye Z, Qian Q (2021) Current progress in CAR-T cell therapy for hematological malignanciess. J Cancer 12(2):326–334 33391429 10.7150/jca.48976 PMC7738987

[128] Perica K, Curran KJ, Brentjens RJ, Giralt SA (2018) Building a CAR garage: preparing for the delivery of commercial CAR T cell products at memorial sloan kettering cancer cente. Biol Blood Marrow Transplant J Am Soc Blood Marrow Transplant 24(6):1135–1141 10.1016/j.bbmt.2018.02.018 PMC6625528 29499327

[129] Haslauer T, Greil R, Zaborsky N, Geisberger R (2021) CAR T-Cell Therapy in hematological malignancies. Int J Mol Sci 22(16). Available from: https://www.mdpi.com/1422-0067/22/16/8996 10.3390/ijms22168996 PMC8396650 34445701

[130] Porter DL, Levine BL, Kalos M, Bagg A, June CH (2011) Chimeric antigen receptor-modified T cells in chronic lymphoid leukemia. N Engl J Med 365(8):725–733 21830940 10.1056/NEJMoa1103849 PMC3387277

[131] Frey NV, Gill S, Hexner EO, Schuster S, Nasta S, Loren A et al (2020) Long-term outcomes from a randomized dose optimization study of chimeric antigen receptor modified T cells in relapsed chronic lymphocytic leukemiaa. J Clin Oncol Off J Am Soc Clin Oncol 38(25):2862–2871 10.1200/JCO.19.03237 PMC8265376 32298202

[132] Pardoll DM (2012) The blockade of immune checkpoints in cancer immunotherapy. Nat Rev Cancer 12(4):252–26 22437870 10.1038/nrc3239 PMC4856023

[133] Sharma P, Allison JP (2015) The future of immune checkpoint therapy. Science 348(6230):56–61 25838373 10.1126/science.aaa8172

[134] Ma X, Edmonson M, Yergeau D, Muzny DM, Hampton OA, Rusch M et al (2015) Rise and fall of subclones from diagnosis to relapse in pediatric B-acute lymphoblastic leukaemia. Nat Commun 6:6604 25790293 10.1038/ncomms7604 PMC4377644

[135] Baeuerle PA, Kufer P, Bargou R (2009) BiTE: Teaching antibodies to engage T-cells for cancer therapy. Curr Opin Mol Ther 11(1):22–30 19169956

[136] Nagorsen D, Baeuerle PA (2011) Immunomodulatory therapy of cancer with T cell-engaging BiTE antibody blinatumomab. Exp Cell Res 317(9):1255–1260 21419116 10.1016/j.yexcr.2011.03.010

[137] Hoffmann P, Hofmeister R, Brischwein K, Brandl C, Crommer S, Bargou R et al (2005) Serial killing of tumor cells by cytotoxic T cells redirected with a CD19-/CD3-bispecific single-chain antibody construct. Int J Cancer 115(1):98–104 15688411 10.1002/ijc.20908

[138] Guo H, Yang J, Wang H, Liu X, Liu Y, Zhou K (2022) Reshaping the tumor microenvironment: The versatility of immunomodulatory drugs in B-cell neoplasms. Front Immunol 13:1017990 36311747 10.3389/fimmu.2022.1017990 PMC9596992

[139] Haslett PA, Corral LG, Albert M, Kaplan G (1998) Thalidomide costimulates primary human T lymphocytes, preferentially inducing proliferation, cytokine production, and cytotoxic responses in the CD8+ subset. J Exp Med 187(11):1885–1892 9607928 10.1084/jem.187.11.1885 PMC2212313

[140] Kater AP, Tonino SH, Egle A, Ramsay AG (2014) How does lenalidomide target the chronic lymphocytic leukemia microenvironment? Blood 124(14):2184–2189 25161268 10.1182/blood-2014-05-578286

[141] Jan M, Sperling AS, Ebert BL (2021) Cancer therapies based on targeted protein degradation - lessons learned with lenalidomide. Nat Rev Clin Oncol 18(7):401–417 33654306 10.1038/s41571-021-00479-z PMC8903027

[142] Fecteau JF, Corral LG, Ghia EM, Gaidarova S, Futalan D, Bharati IS et al (2014) Lenalidomide inhibits the proliferation of CLL cells via a cereblon/p21(WAF1/Cip1)-dependent mechanism independent of functional p53. Blood 124(10):1637–1644 24990888 10.1182/blood-2014-03-559591 PMC4155272

[143] Ramsay AG, Evans R, Kiaii S, Svensson L, Hogg N, Gribben JG (2013) Chronic lymphocytic leukemia cells induce defective LFA-1-directed T-cell motility by altering Rho GTPase signaling that is reversible with lenalidomide. Blood 121(14):2704–2714 23325833 10.1182/blood-2012-08-448332 PMC3617635

[144] Styczynski J, Czyzewski K, Wysocki M (2006) Ex vivo activity of thalidomide in childhood acute leukemia. Leuk Lymphoma 47(6):1123–1128 16840205 10.1080/10428190500467891

[145] Chen GS, Yu HS, Lan CCE, Chow KC, Lin TY, Kok LF et al (2006) CXC chemokine receptor CXCR4 expression enhances tumorigenesis and angiogenesis of basal cell carcinoma. Br J Dermatol 154(5):910–918 16634895 10.1111/j.1365-2133.2006.07150.x

[146] Stamatopoulos B, Meuleman N, De Bruyn C, Pieters K, Mineur P, Le Roy C et al (2012) AMD3100 disrupts the cross-talk between chronic lymphocytic leukemia cells and a mesenchymal stromal or nurse-like cell-based microenvironment: pre-clinical evidence for its association with chronic lymphocytic leukemia treatments. Haematologica 97(4):608–615 22058221 10.3324/haematol.2011.052779 PMC3347654

[147] Kashyap MK, Amaya-Chanaga CI, Kumar D, Simmons B, Huser N, Gu Y et al (2017) Targeting the CXCR4 pathway using a novel anti-CXCR4 IgG1 antibody (PF-06747143) in chronic lymphocytic leukemia. J Hematol Oncol 10(1):112 28526063 10.1186/s13045-017-0435-x PMC5438492

[148] Stamatopoulos B, Meuleman N, De Bruyn C, Delforge A, Bron D, Lagneaux L (2010) The histone deacetylase inhibitor suberoylanilide hydroxamic acid induces apoptosis, down-regulates the CXCR4 chemokine receptor and impairs migration of chronic lymphocytic leukemia cells. Haematologica 95(7):1136–1143 20145270 10.3324/haematol.2009.013847 PMC2895038

[149] Juarez J, Dela Pena A, Baraz R, Hewson J, Khoo M, Cisterne A et al (2007) CXCR4 antagonists mobilize childhood acute lymphoblastic leukemia cells into the peripheral blood and inhibit engraftment. Leukemia 21(6):1249–1257 17410186 10.1038/sj.leu.2404684

[150] Kapp TG, Rechenmacher F, Sobahi TR, Kessler H (2013) Integrin modulators: a patent review. Expert Opin Ther Pat 23(10):1273–1295 24050747 10.1517/13543776.2013.818133

[151] Hsieh YT, Gang EJ, Shishido SN, Kim HN, Pham J, Khazal S et al (2014) Effects of the small-molecule inhibitor of integrin α4, TBC3486, on pre-B-all cells. Leukemia, England (Vol. 28 pp 2101–2104) 10.1038/leu.2014.182 PMC4190402 24903479

[152] Muz B, de la Puente P, Azab F, Luderer M, Azab AK (2014) The role of hypoxia and exploitation of the hypoxic environment in hematologic malignancies. Mol Cancer Res 12(10):1347–1354 25158954 10.1158/1541-7786.MCR-14-0028

[153] Benito J, Shi Y, Szymanska B, Carol H, Boehm I, Lu H et al (2011) Pronounced hypoxia in models of murine and human leukemia: high efficacy of hypoxia-activated prodrug PR-104. PLoS ONE 6(8):e23108 21853076 10.1371/journal.pone.0023108 PMC3154919

[154] Herman SEM, Gordon AL, Hertlein E, Ramanunni A, Zhang X, Jaglowski S et al (2011) Bruton tyrosine kinase represents a promising therapeutic target for treatment of chronic lymphocytic leukemia and is effectively targeted by PCI-32765. Blood 117(23):6287–6296 21422473 10.1182/blood-2011-01-328484 PMC3122947

[155] O’Brien S, Furman RR, Coutre S, Flinn IW, Burger JA, Blum K et al (2018) Single-agent ibrutinib in treatment-naïve and relapsed/refractory chronic lymphocytic leukemia: a 5-year experience. Blood 131(17):1910–1919 29437592 10.1182/blood-2017-10-810044 PMC5921964

[156] Burger JA, Buggy JJ (2013) Bruton tyrosine kinase inhibitor ibrutinib (PCI-32765). Leuk Lymphoma 54(11):2385–2391 23425038 10.3109/10428194.2013.777837

[157] Ponader S, Chen SS, Buggy JJ, Balakrishnan K, Gandhi V, Wierda WG et al (2012) The Bruton tyrosine kinase inhibitor PCI-32765 thwarts chronic lymphocytic leukemia cell survival and tissue homing in vitro and in vivo. Blood 119(5):1182–1189 22180443 10.1182/blood-2011-10-386417 PMC4916557

[158] Sharman JP, Egyed M, Jurczak W, Skarbnik A, Pagel JM, Flinn IW et al (2020) Acalabrutinib with or without obinutuzumab versus chlorambucil and obinutuzmab for treatment-naive chronic lymphocytic leukaemia (ELEVATE TN): a randomised, controlled, phase 3 trial. Lancet (London, England) 395(10232):1278–1291 32305093 10.1016/S0140-6736(20)30262-2 PMC8151619

[159] Gomez EB, Isabel L, Rosendahal MS, Rothenberg SM, Andrews SW, Brandhuber BJ (2019) Loxo-305, a highly selective and non-covalent next generation BTK inhibitor, inhibits diverse BTK C481 substitution mutations. Blood 134(Supplement_1):4644. 10.1182/blood-2019-126114

[160] Mato AR, Shah NN, Jurczak W, Cheah CY, Pagel JM, Woyach JA et al (2021) Pirtobrutinib in relapsed or refractory B-cell malignancies (BRUIN): a phase 1/2 study. Lancet (London, England) 397(10277):892–901 33676628 10.1016/S0140-6736(21)00224-5 PMC11758240

[161] Aslan B, Kismali G, Iles LR, Manyam GC, Ayres ML, Chen LS et al (2022) Pirtobrutinib inhibits wild-type and mutant Bruton’s tyrosine kinase-mediated signaling in chronic lymphocytic leukemia. Blood Cancer J 12(5):80 35595730 10.1038/s41408-022-00675-9 PMC9123190

[162] Lannutti BJ, Meadows SA, Herman SEM, Kashishian A, Steiner B, Johnson AJ et al (2011) CAL-101, a p110delta selective phosphatidylinositol-3-kinase inhibitor for the treatment of B-cell malignancies, inhibits PI3K signaling and cellular viability. Blood 117(2):591–594 20959606 10.1182/blood-2010-03-275305 PMC3694505

[163] Ikeda H, Hideshima T, Fulciniti M, Perrone G, Miura N, Yasui H et al (2010) PI3K/p110{delta} is a novel therapeutic target in multiple myeloma. Blood 116(9):1460–1468 20505158 10.1182/blood-2009-06-222943 PMC2938837

[164] Brown JR, Byrd JC, Coutre SE, Benson DM, Flinn IW, Wagner-Johnston ND et al (2014) Idelalisib, an inhibitor of phosphatidylinositol 3-kinase p110δ, for relapsed/refractory chronic lymphocytic leukemia. Blood 123(22):3390–3397 24615777 10.1182/blood-2013-11-535047 PMC4123414

[165] Flinn IW, Hillmen P, Montillo M, Nagy Z, Illés Á, Etienne G et al (2018) The phase 3 DUO trial: duvelisib vs ofatumumab in relapsed and refractory CLL/SLL. Blood 132(23):2446–2455 30287523 10.1182/blood-2018-05-850461 PMC6284216

[166] Stephens DM, Huang Y, Ruppert AS, Walker JS, Canfield D, Cempre CB et al (2022) Selinexor combined with ibrutinib demonstrates tolerability and safety in advanced B-cell malignancies: a phase I study. Clin Cancer Res an Off J Am Assoc Cancer Res 28(15):3242–3247 10.1158/1078-0432.CCR-21-3867 PMC9364840 35608822

[167] Weinblatt ME, Kavanaugh A, Burgos-Vargas R, Dikranian AH, Medrano-Ramirez G, Morales-Torres JL et al (2008) Treatment of rheumatoid arthritis with a Syk kinase inhibitor: a twelve-week, randomized, placebo-controlled trial. Arthritis Rheum 58(11):3309–3318 18975322 10.1002/art.23992

[168] Braselmann S, Taylor V, Zhao H, Wang S, Sylvain C, Baluom M et al (2006) R406, an orally available spleen tyrosine kinase inhibitor blocks fc receptor signaling and reduces immune complex-mediated inflammation. J Pharmacol Exp Ther 319(3):998–1008 16946104 10.1124/jpet.106.109058

[169] Paiva C, Rowland TA, Sreekantham B, Godbersen C, Best SR, Kaur P et al (2017) SYK inhibition thwarts the BAFF - B-cell receptor crosstalk and thereby antagonizes Mcl-1 in chronic lymphocytic leukemia. Haematologica 102(11):1890–1900 28838991 10.3324/haematol.2017.170571 PMC5664393

[170] Advani RH, Buggy JJ, Sharman JP, Smith SM, Boyd TE, Grant B et al (2013) Bruton tyrosine kinase inhibitor ibrutinib (PCI-32765) has significant activity in patients with relapsed/refractory B-cell malignancies. J Clin Oncol Off J Am Soc Clin Oncol 31(1):88–94 10.1200/JCO.2012.42.7906 PMC5505166 23045577

[171] Ruan Y, Kim HN, Ogana H, Kim YM (2020) Wnt signaling in leukemia and its bone marrow microenvironment. Int J Mol Sci 21(17) 10.3390/ijms21176247 PMC7503842 32872365

[172] Duque-Afonso J, Lin CH, Han K, Morgens DW, Jeng EE, Weng Z et al (2018) CBP modulates sensitivity to dasatinib in Pre-BCR(+) acute lymphoblastic leukemia. Cancer Res 78(22):6497–6508 30262461 10.1158/0008-5472.CAN-18-1703 PMC6283070

[173] Kim YM, Gang EJ, Kahn M (2017) CBP/Catenin antagonists: Targeting LSCs’ Achilles heel. Exp Hematol 52:1–11 28479420 10.1016/j.exphem.2017.04.010 PMC5526056

[174] Choi MY, Widhopf GF 2nd, Wu CCN, Cui B, Lao F, Sadarangani A et al (2015) Pre-clinical specificity and safety of UC-961, a first-in-class monoclonal antibody targeting ROR1. Clin Lymphoma Myeloma Leuk 15 Suppl(0):S167–169 10.1016/j.clml.2015.02.010 PMC4548279 26297272

[175] Coleman RL, Handley KF, Burger R, Molin GZD, Stagg R, Sood AK et al (2020) Demcizumab combined with paclitaxel for platinum-resistant ovarian, primary peritoneal, and fallopian tube cancer: The SIERRA open-label phase Ib trial. Gynecol Oncol 157(2):386–391 32037195 10.1016/j.ygyno.2020.01.042

[176] Ferrarotto R, Eckhardt G, Patnaik A, LoRusso P, Faoro L, Heymach JV et al (2018) A phase I dose-escalation and dose-expansion study of brontictuzumab in subjects with selected solid tumors. Ann Oncol Off J Eur Soc Med Oncol 29(7):1561–1568 10.1093/annonc/mdy171 29726923

[177] Fraietta JA, Beckwith KA, Patel PR, Ruella M, Zheng Z, Barrett DM et al (2016) Ibrutinib enhances chimeric antigen receptor T-cell engraftment and efficacy in leukemia. Blood 127(9):1117–1127 26813675 10.1182/blood-2015-11-679134 PMC4778162

[178] Gang EJ, Hsieh YT, Pham J, Zhao Y, Nguyen C, Huantes S et al (2014) Small-molecule inhibition of CBP/catenin interactions eliminates drug-resistant clones in acute lymphoblastic leukemia. Oncogene 33(17):2169–2178 23728349 10.1038/onc.2013.169 PMC3994178

[179] Wu W, Zhu H, Fu Y, Shen W, Miao K, Hong M et al (2016) High LEF1 expression predicts adverse prognosis in chronic lymphocytic leukemia and may be targeted by ethacrynic acid. Oncotarget 7(16):21631–21643 26950276 10.18632/oncotarget.7795 PMC5008311

[180] Janovská P, Bryja V (2017) Wnt signalling pathways in chronic lymphocytic leukaemia and B-cell lymphomas. Br J Pharmacol 174(24):4701–4715 28703283 10.1111/bph.13949 PMC5727250

[181] Meng QJ, Maywood ES, Bechtold DA, Lu WQ, Li J, Gibbs JE et al (2010) Entrainment of disrupted circadian behavior through inhibition of casein kinase 1 (CK1) enzymes. Proc Natl Acad Sci U S A 107(34):15240–15245 20696890 10.1073/pnas.1005101107 PMC2930590

[182] Arey R, McClung CA (2012) An inhibitor of casein kinase 1 ε/δ partially normalizes the manic-like behaviors of the ClockΔ19 mouse. Behav Pharmacol 23(4):392–396 22743604 10.1097/FBP.0b013e32835651fd PMC3673712

[183] Cheong JK, Virshup DM (2016) CK1δ: a pharmacologically tractable Achilles’ heel of Wnt-driven cancers? Ann Transl Med 4(21):433 27942524 10.21037/atm.2016.11.07 PMC5124623

[184] Daneshmanesh AH, Hojjat-Farsangi M, Khan AS, Jeddi-Tehrani M, Akhondi MM, Bayat AA et al (2012) Monoclonal antibodies against ROR1 induce apoptosis of chronic lymphocytic leukemia (CLL) cells. Leukemia 26(6):1348–1355 22289919 10.1038/leu.2011.362

[185] Hudecek M, Schmitt TM, Baskar S, Lupo-Stanghellini MT, Nishida T, Yamamoto TN et al (2010) The B-cell tumor-associated antigen ROR1 can be targeted with T cells modified to express a ROR1-specific chimeric antigen receptor. Blood 116(22):4532–4541 20702778 10.1182/blood-2010-05-283309 PMC2996114

[186] Takebe N, Nguyen D, Yang SX (2014) Targeting notch signaling pathway in cancer: clinical development advances and challenges. Pharmacol Ther 141(2):140–149 24076266 10.1016/j.pharmthera.2013.09.005 PMC3982918

[187] Wu Y, Cain-Hom C, Choy L, Hagenbeek TJ, de Leon GP, Chen Y et al (2010) Therapeutic antibody targeting of individual Notch receptors. Nature 464(7291):1052–1057 20393564 10.1038/nature08878

[188] Minuzzo S, Agnusdei V, Pusceddu I, Pinazza M, Moserle L, Masiero M et al (2015) DLL4 regulates NOTCH signaling and growth of T acute lymphoblastic leukemia cells in NOD/SCID mice. Carcinogenesis 36(1):115–121 25355291 10.1093/carcin/bgu223

